# Long-Distance Q-Resolution with Dependency Schemes

**DOI:** 10.1007/s10817-018-9467-3

**Published:** 2018-06-09

**Authors:** Tomáš Peitl, Friedrich Slivovsky, Stefan Szeider

**Affiliations:** Algorithms and Complexity Group, TU Wien, 1040 Vienna, Austria

**Keywords:** QBF, Q-resolution, Dependency schemes, Strategy extraction

## Abstract

Resolution proof systems for quantified Boolean formulas (QBFs) provide a formal model for studying the limitations of state-of-the-art search-based QBF solvers that use these systems to generate proofs. We study a combination of two proof systems supported by the solver DepQBF: Q-resolution with generalized universal reduction according to a dependency scheme and long distance Q-resolution. We show that the resulting proof system—which we call long-distance Q(D)-resolution—is sound for the reflexive resolution-path dependency scheme. In fact, we prove that it admits strategy extraction in polynomial time. This comes as an application of a general result, by which we identify a whole class of dependency schemes for which long-distance Q(D)-resolution admits polynomial-time strategy extraction. As a special case, we obtain soundness and polynomial-time strategy extraction for long distance Q(D)-resolution with the standard dependency scheme. We further show that search-based QBF solvers using a dependency scheme D and learning with long-distance Q-resolution generate long-distance Q(D)-resolution proofs. The above soundness results thus translate to partial soundness results for such solvers: they declare an input QBF to be false only if it is indeed false. Finally, we report on experiments with a configuration of DepQBF that uses the standard dependency scheme and learning based on long-distance Q-resolution.

## Introduction

Quantified Boolean formulas (QBFs) offer succinct encodings for problems from domains such as formal verification, synthesis, and planning [5, 13, 16, 30, 38, 43]. Although the combination of (more verbose) propositional encodings with SAT solvers is still the state-of-the-art approach to many of these problems, QBF solvers are gaining ground. An arsenal of new techniques has been introduced over the past few years [10, 11, 14, 22, 23, 25, 26, 29, 32, 33, 35], and these advances in solver technology have been accompanied by the development of a better understanding of the underlying QBF proof systems and their limitations [4, 7–9, 12, 18, 27, 42].

Search-based solvers implementing the QCDCL algorithm [15, 46] represent one of the principal state-of-the-art approaches in QBF solving. Akin to modern SAT solvers, these solvers rely on successive variable assignments in combination with fast constraint propagation and learning. Unlike SAT solvers, however, search-based QBF solvers are constrained by the variable dependencies induced by the quantifier prefix:^1^ while SAT solvers can assign variables in any order, search-based QBF solvers can only assign variables from the leftmost quantifier block that contains unassigned variables, since the assignment of a variable further to the right might depend on the variable assignment to this block. In the most extreme case, this forces solvers into a fixed order of variable assignments, rendering decision variable heuristics ineffective.

The search-based solver DepQBF uses dependency schemes to partially bypass this restriction [10, 31]. Dependency schemes can sometimes identify pairs of variables as independent, allowing the solver to assign them in any order. This gives decision heuristics more freedom and results in increased performance [10].

While this provides a strong motivation to use dependency schemes, their integration with QCDCL poses challenges of its own. Soundness of the proof system underlying QCDCL with the standard dependency scheme as implemented in DepQBF was shown only recently [42], and combining other state-of-the-art techniques with dependency schemes is often highly nontrivial. In this paper, we focus on two such issues:

- Long-distance Q-resolution permits the derivation of tautological clauses in certain cases [2, 45, 47]. This system can be used in constraint learning as an alternative to Q-resolution, leading to fewer backtracks during search and, sometimes, reduced runtime [19]. In addition, clause learning based on long-distance Q-resolution is substantially easier to implement. However, it is open whether QCDCL with learning based on long-distance Q-resolution is sound when combined with known dependency schemes.
- For applications in verification and synthesis, it is not enough for solvers to decide whether an input QBF is true or false—they also have to generate a certificate. Such certificates can be efficiently constructed from Q-resolution [2] and even long-distance Q-resolution proofs [3]. However, it is not clear whether this is possible for proofs generated by QCDCL with the standard dependency scheme.

We define LDQ(D)-resolution as consisting of long-distance Q-resolution with a dependency scheme D, and show that a search-based QBF solver using dependency scheme D and learning based on long-distance Q-resolution generates an LDQ(D)-resolution refutation whenever it declares an input QBF to be false. This allows us to partially address (a) by showing that long-distance Q-resolution combined with the reflexive resolution-path dependency scheme [42] is sound. In fact, we prove that this proof system allows for certificate extraction in polynomial time, thus resolving (b) as well. These results also hold for long-distance Q-resolution combined with the weaker standard dependency scheme. We thus provide a partial soundness argument for QCDCL with these dependency schemes and learning based on long-distance Q-resolution to the effect that “false” answers can be trusted.

Our proof relies on a familiar interpretation of Q-resolution refutations as winning strategies for the universal player in the evaluation game [24]. We identify a natural property of dependency schemes D that not only allows for the interpretation of an LDQ(D)-refutation as a winning strategy for the universal player, but even implies polynomial-time certificate extraction from an LDQ(D)-refutation. We then show that the reflexive resolution path dependency scheme in fact has this property.

One of our motivations for studying the combination of long-distance Q-resolution and dependency schemes is that it is already supported by DepQBF. To complement our theoretical results, and to provide further motivation for resolving the question of soundness of “true” answers, we performed experiments using a configuration of DepQBF with both features activated. These experiments show that performance with learning based on LDQ(D)-resolution is on par with and—in some cases—even slightly better than the performance of DepQBF with other configurations of constraint learning.

### Organization

Section 2 establishes basic notions used throughout this paper. In Sect. 3, we review dependency schemes and introduce the LDQ(D) proof system. In Sect. 4, we present a version of QCDCL which combines dependency schemes with learning based on long-distance Q-resolution and argue that this algorithm generates LDQ(D) proofs. Section 5 is split into two parts: in the first part, we define a property of dependency schemes D and prove that it is sufficient for soundness of LDQ(D); in the second part, we show that the reflexive resolution-path dependency scheme has this property. In Sect. 6, we report on experiments with a version of DepQBF that generates LDQ(D$^\text {std}$)-proofs. In Sect. 7, we briefly discuss recently published related work. We conclude in Sect. 8 with some open questions.

## Preliminaries

###  Formulas and Assignments

A *literal* is a negated or unnegated variable. If *x* is a variable, we write $$\overline{x} = \lnot x$$ and $$\overline{\lnot x} = x$$, and let $$ var (x) = var (\lnot x) = x$$. We sometimes call literals *x* and $$\lnot x$$ the positive and negative *polarity* of variable *x*. If *X* is a set of literals, we write $$\overline{X}$$ for the set $$\{\,\overline{x} \;{:}\;x \in X \,\}$$. A *clause* is a finite disjunction of literals, and a *term* is a finite conjunction of literals. We call a clause *tautological* if it contains the same variable negated as well as unnegated. A *CNF formula* is a finite conjunction of non-tautological clauses. Whenever convenient, we treat clauses and terms as sets of literals, and CNF formulas as sets of sets of literals. We write $$ var (S)$$ for the set of variables occurring (negated or unnegated) in a clause or term *S*, that is, $$ var (S) = \{\, var (\ell ) \;{:}\;\ell \in S \,\}$$. Moreover, we let $$ var (\varphi ) = \bigcup _{C \in \varphi } var (C)$$ denote the set of variables occurring in a CNF formula $$\varphi $$.

A *truth assignment* (or simply *assignment*) to a set *X* of variables is a mapping $\tau : X \rightarrow \{0,1\}$. We write [*X*] for the set of truth assignments to *X*, and extend $\tau : X \rightarrow \{0, 1\}$ to literals by letting $\tau (\lnot x) = 1 - \tau (x)$ for $x \in X$. Let $\tau : X \rightarrow \{0, 1\}$ be a truth assignment. The restriction $C[\tau ]$ of a clause (term) *S* by $\tau$ is defined as follows: if there is a literal $\ell \in S \cap (X \cup \overline{X})$ such that $\tau (\ell ) = 1$ ($\tau (\ell ) = 0)$ then $S[\tau ] = 1$ ($S[\tau ] = 0$). Otherwise, $S[\tau ] = S {\setminus } (X \cup \overline{X})$. The restriction $\varphi [\tau ]$ of a CNF formula $\varphi$ by the assignment $\tau$ is defined $\varphi [\tau ] = \{\,C[\tau ] \;{:}\;C \in \varphi , C[\tau ] \ne 1 \,\}$.

### PCNF Formulas

A *PCNF formula* is denoted by $\varPhi = {\mathcal {Q}}.\varphi$, where $\varphi$ is a CNF formula and $\mathcal {Q}= Q_1 X_1 \dots Q_n X_n$ is a sequence such that $Q_i \in \{\forall , \exists \}$, $Q_i \ne Q_{i+1}$ for $1 \le i < n$, and the $X_i$ are pairwise disjoint sets of variables. We call $\varphi$ the *matrix* of $\varPhi$ and $\mathcal {Q}$ the *(quantifier) prefix* of $\varPhi$, and refer to the $X_i$ as *quantifier blocks*. We require that $var (\varphi ) = X_1 \cup \dots \cup X_n$ and write $var (\varPhi ) = var (\varphi )$. We define a partial order $<_{\varPhi }$ on $var (\varphi )$ as $x<_{\varPhi } y \Leftrightarrow x \in X_i, y \in X_j, i < j$. We extend $<_{\varPhi }$ to a relation on literals in the obvious way and drop the subscript whenever $\varPhi$ is understood. For $x \in var (\varPhi )$ we let $R_{\varPhi }(x) = \{\,y \in var (\varPhi ) \;{:}\;x <_{\varPhi } y \,\}$ and $L_{\varPhi }(x) = \{\,y \in var (\varPhi ) \;{:}\;y <_{\varPhi } x \,\}$ denote the sets of variables *to the right* and *to the left* of *x* in $\varPhi$, respectively. Relative to the PCNF formula $\varPhi$, variable *x* is called *existential* (*universal*) if $x \in X_i$ and $Q_i = \exists$ ($Q_i = \forall$). The set of existential (universal) variables occurring in $\varPhi$ is denoted $var _{\exists }(\varPhi )$ ($var _{\forall }(\varPhi )$). The *size* of a PCNF formula $\varPhi = {\mathcal {Q}}.\varphi$ is defined as $|\varPhi | = \sum _{C \in \varphi }|C|$. If $\tau$ is an assignment, then $\varPhi [\tau ]$ denotes the PCNF formula $\mathcal {Q}'.\varphi [\tau ]$, where $\mathcal {Q}'$ is the quantifier prefix obtained from $\mathcal {Q}$ by deleting variables that do not occur in $\varphi [\tau ]$. *True* and *false* PCNF formulas are defined in the usual way.

### Countermodels

Let $\varPhi = \mathcal {Q}.\varphi$ be a PCNF formula. A *countermodel* of $\varPhi$ is an indexed family $\{f_{u}\}_{u \in var _{\forall }(\varPhi )}$ of functions $f_u: [L_{\varPhi }(u)] \rightarrow \{0, 1\}$ such that $\varphi [\tau ] = \{\emptyset \}$ for every assignment $\tau : var (\varPhi ) \rightarrow \{0, 1\}$ satisfying $\tau (u) = f_u(\tau |_{L_{\varPhi }(u)})$ for $u \in var _{\forall }(\varPhi )$.

#### Proposition 1

(Folklore) A PCNF formula is false if, and only if, it has a countermodel.

## Dependency Schemes and LDQ(D)-Resolution

In this section, we introduce the proof system LDQ(D), which combines Q(D)-resolution [42] with long-distance Q-resolution [2]. Q-resolution is a generalization of propositional resolution to PCNF formulas [28]. Q-resolution is of practical interest due to its relation to search based QBF solvers that implement Quantified Conflict Driven Constraint Learning (QCDCL) [15, 46]: the traces of QCDCL solvers correspond to Q-resolution proofs [19, 21]. QCDCL—which will be described in more detail in Sect. 4—generalizes the well-known DPLL procedure [17] from SAT to QSAT. In a nutshell, DPLL searches for a satisfying assignment of an input formula by propagating unit clauses and assigning pure literals until the formula cannot be simplified any further, at which point it picks an unassigned variable and branches on the assignment of this variable. Any of the remaining variables can be chosen for assignment, but the order of assignment can have significant effects on the runtime. Modern conflict driven clause learning (CDCL) SAT solvers derived from the DPLL algorithm use sophisticated heuristics to determine what variable to assign next [34].

In QCDCL, the quantifier prefix imposes constraints on the order of variable assignments: a variable may be assigned only if it occurs in the leftmost quantifier block with unassigned variables. Often, this is more restrictive than necessary. For instance, variables from disjoint subformulas may be assigned in any order. Intuitively, a variable can be assigned as long as it *does not depend* on any unassigned variable. This is the intuition underlying a generalization of QCDCL implemented in the solver DepQBF [10, 31]. DepQBF uses a *dependency scheme* [39] to compute an overapproximation of variable dependencies. Dependency schemes are mappings that associate every PCNF formula with a binary relation on its variables that refines the order of variables in the quantifier prefix.^2^

### Definition 1

(*Dependency Scheme*) A *dependency scheme* is a mapping *D* that associates each PCNF formula $\varPhi$ with a relation $D_{\varPhi } \subseteq \{\,(x, y) \;{:}\;x <_{\varPhi } y \,\}$ called the *dependency relation* of $\varPhi$ with respect to *D*.

The mapping which simply returns the prefix ordering of an input formula can be thought of as a baseline dependency scheme:

### Definition 2

(*Trivial Dependency Scheme*) The *trivial dependency scheme*$\text {D}^{\text { trv}}$ associates each PCNF formula $\varPhi$ with the relation $\text {D}^{\text { trv}}_{\varPhi } = \{\,(x, y) \;{:}\;x <_{\varPhi } y \,\}$.

DepQBF uses a dependency relation to determine the order in which variables can be assigned: if *y* is a variable and there is no unassigned variable *x* such that (*x*, *y*) is in the dependency relation, then *y* is considered ready for assignment. DepQBF also uses the dependency relation to generalize the $\forall$-reduction rule used in clause learning [10]. As a result of its use of dependency schemes, DepQBF generates proofs in a generalization of Q-resolution called Q(D)-resolution [42], a proof system that takes a dependency scheme D as a parameter.

Dependency schemes can be partially ordered based on their dependency relations: if the dependency relation computed by a dependency scheme $D_1$ is a subset of the dependency relation computed by a dependency scheme $D_2$ for each PCNF formula, then $D_1$ is *more general* than $D_2$. The more general a dependency scheme, the more freedom DepQBF has in choosing decision variables. Currently, (aside from the trivial dependency scheme) DepQBF supports the so-called *standard dependency scheme* [39].^3^ We will work with the more general *reflexive resolution-path dependency scheme* [42], a variant of the resolution-path dependency scheme [41, 44]. This dependency scheme computes an overapproximation of variable dependencies based on whether two variables are connected by a (pair of) resolution path(s).

### Definition 3

(*Resolution Path*) Let $\varPhi = {\mathcal {Q}}.\varphi$ be a PCNF formula and let *X* be a set of variables. A *resolution path* (from $\ell _1$ to $\ell _{2k}$) via *X* (in $\varPhi$) is a sequence $\ell _1, \ldots , \ell _{2k}$ of literals satisfying the following properties:

1. For all $i \in [k]$, there is a $C_i \in \varphi$ such that $\ell _{2i-1}, \ell _{2i} \in C_i$.
2. For all $$i \in [k]$$, $$ var (\ell _{2i-1}) \ne var (\ell _{2i})$$.
3. For all $$i \in [k-1]$$, $$\{\ell _{2i}, \ell _{2i+1}\} \subseteq X \cup \overline{X}$$.
4. For all $$i \in [k-1]$$, $$\overline{\ell _{2i}} = \ell _{2i+1}$$.

If $\pi = \ell _1, \ldots , \ell _{2k}$ is a resolution path in $\varPhi$ via *X*, we say that $\ell _1$ and $\ell _{2k}$ are *connected in*$\varPhi$ (with respect to *X*). For every $i \in \{1, \ldots , k-1\}$ we say that $\pi$*goes through*$var (\ell _{2i})$.

One can think of a resolution path as a potential chain of implications: if each clause $C_i$ contains exactly two literals, then assigning $\ell _1$ to 0 requires setting $\ell _{2k}$ to 1. If, in addition, there is such a path from $\overline{\ell _1}$ to $\overline{\ell _{2k}}$, then $\ell _1$ and $\ell _{2k}$ have to be assigned opposite values. Accordingly, the resolution path dependency scheme identifies variables connected by a pair of resolution paths as potentially dependent on each other.

### Definition 4

(*Dependency Pair*) Let $\varPhi$ be a PCNF formula and $x, y \in var (\varPhi )$. We say $\{x,y\}$ is a *resolution-path dependency pair* of $\varPhi$ with respect to $X \subseteq var _{\exists }(\varPhi )$ if at least one of the following conditions holds:

- *x* and *y*, as well as $\lnot x$ and $\lnot y$, are connected in $\varPhi$ with respect to *X*.
- *x* and $\lnot y$, as well as $\lnot x$ and *y*, are connected in $\varPhi$ with respect to *X*.

### Definition 5

The *reflexive resolution-path dependency scheme* is the mapping $\text { D }^{\text { rrs}}$ that assigns to each PCNF formula $\varPhi = {\mathcal {Q}}.\varphi$ the relation $\text { D }^{\text { rrs}}_{\varPhi } = \{\,x <_{\varPhi } y \;{:}\;\{x,y\}$ is a resolution-path dependency pair in $\varPhi$ with respect to $R_{\varPhi }(x) {\setminus } var _{\forall }(\varPhi ) \,\}$.

Both Q-resolution and Q(D)-resolution only allow for the derivation of non-tautological clauses, that is, clauses that do not contain a literal negated as well as unnegated. *Long-distance Q-resolution* is a variant of Q-resolution that admits tautological clauses in certain cases [2]. Variants of QCDCL that allow for learned clauses to be tautological [45, 47] have been shown to generate proofs in long-distance Q-resolution [19].

**Fig. 1:**
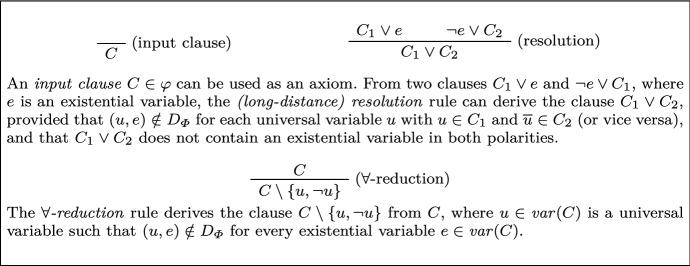
Derivation rules of LDQ(D)-resolution for a PCNF formula $\varPhi =\mathcal {Q}. \varphi$

In long-distance Q-resolution, when a tautological clause is created by resolution, a variable that appears in both polarities must be to the right of the pivot variable. We generalize this by requiring that the pivot be independent of a tautological variable to obtain *long-distance Q(D)-resolution* (LDQ(D)-resolution). The derivation rules of LDQ(D)-resolution are shown in Fig. 1.^4^ Here, as in the rest of the paper, D denotes an arbitrary dependency scheme.

A derivation in a proof system consists of repeated applications of the derivation rules to derive a clause from the clauses of an input formula. Here, derivations will be represented by node-labeled directed acyclic graphs (DAGs). More specifically, we require these DAGs to have a unique sink (that is, a node without outgoing edges) and each of their nodes to have at most two incoming edges. We further assume an ordering on the in-neighbors (or parents) of every node with two incoming edges—that is, each node has a “first” and a “second” in-neighbor. Referring to such DAGs as *proof DAGs*, we define the following two operations to represent resolution and $\forall$-reduction:

1. If $\ell$ is a literal and $\mathcal {P}_1$ and $\mathcal {P}_2$ are proof DAGs with distinct sinks $v_1$ and $v_2$, then $\mathcal {P}_1 \odot _{\ell } \mathcal {P}_2$ is the proof DAG consisting of the union of $\mathcal {P}_1$ and $\mathcal {P}_2$ along with a new sink *v* that has two incoming edges, the first one from $v_1$ and the second one from $v_2$. Moreover, if $C_1$ is the label of $v_1$ in $\mathcal {P}_1$ and $C_2$ is the label of $v_2$ in $\mathcal {P}_2$, then *v* is labeled with the clause $(C_1 {\setminus } \{\ell \}) \cup (C_2 {\setminus } \{\overline{\ell }\})$.
2. If *u* is a variable and $\mathcal {P}$ is a proof DAG with a sink *w* labeled with *C*, then $\mathcal {P}- u$ denotes the proof DAG obtained from $\mathcal {P}$ by adding an edge from *w* to a new node *v* such that *v* is labeled with $C {\setminus } \{u, \lnot u\}$.

### Definition 6

(*Derivation*) An *LDQ(D)-resolution derivation* (*LDQ(D)-derivation* for short) of a clause *C* from a PCNF formula $\varPhi = \mathcal {Q}.\varphi$ is a proof DAG $\mathcal {P}$ satisfying the following properties.

- Source nodes are labeled with input clauses from $\varphi$.
- If a node with label *C* has parents labeled $C_1$ and $C_2$ then *C* can be derived from $C_1$ and $C_2$ by (long-distance) resolution.
- If a node labeled with a clause *C* has a single parent with label $C'$ then *C* can be derived from $C'$ by $\forall$-reduction with respect to the dependency scheme D.

We refer to these nodes as *input nodes*, *resolution nodes*, and $\forall$*-reduction nodes*, respectively.

Let $\mathcal {P}$ be an LDQ(D)-derivation from a PCNF formula $\varPhi$. The (clause) label of the sink node is called the *conclusion* of $\mathcal {P}$, denoted $Cl(\mathcal {P})$. If the conclusion of $\mathcal {P}$ is the empty clause then we refer to $\mathcal {P}$ as an *LDQ(D)-refutation* of $\varPhi$. For a node *v* of $\mathcal {P}$, the *subderivation* (of $\mathcal {P}$) rooted at *v* is the proof DAG induced by *v* and its ancestors in $\mathcal {P}$. It is straightforward to verify that the resulting proof DAG is again an LDQ(D)-derivation from $\varPhi$. For convenience, we will identify (sub)derivations with their sinks. The *size* of $\mathcal {P}$, denoted $|\mathcal {P}|$, is the total number of literal occurrences in clause labels of $\mathcal {P}$.

## QCDCL with Dependency Schemes Generates LDQ(D)-Proofs

In this section, we present a version of the QCDCL algorithm that uses dependency schemes [10, 31] and performs constraint learning based on long-distance Q-resolution [19, 46].^5^ Generalizing an argument by Egly et al.  [19], we will show that this algorithm produces LDQ(D)-proofs when using a dependency scheme D.

Starting from an input PCNF formula $\varPhi$, QCDCL generates (“learns”) constraints—clauses and terms—until it produces an empty constraint, at which point it returns true (if the empty term is learned) or false (if the empty clause is learned).

One can think of QCDCL solving as proceeding in rounds. Along with a set of clauses and terms, the solver maintains an assignment $\sigma$ (the *trail*, which we will represent by a sequence of literals in the order of their assignment). During each round, this assignment is extended by quantified Boolean constraint propagation (QBCP) and—possibly—branching.

*Quantified Boolean constraint propagation* (with dependency scheme D) consists in the exhaustive application of universal and existential reduction (relative to $D_{\varPhi }$) in combination with unit assignments.^6^ More specifically, QBCP reports a clause *C* as falsified if $C[\sigma ] \ne 1$ and universal reduction can be applied to $C[\sigma ]$ to obtain the empty clause. Dually, a term *T* is considered satisfied if $T[\sigma ] \ne 0$ and $T[\sigma ]$ can be reduced to the empty term. A clause *C* is *unit* under $\sigma$ if $C[\sigma ] \ne 1$ and universal reduction yields a clause $C' = (\ell )$, for some existential literal $\ell$ such that $var (\ell )$ is unassigned. Dually, a term *T* is *unit* under $\sigma$ if $T[\sigma ] \ne 0$ and existential reduction can be applied to obtain a term $T' = (\ell )$ containing a single universal literal $\ell$. If $C = (\ell )$ is a unit clause, then the assignment $\sigma$ has to be extended by $\ell$ in order not to falsify *C*, and if $T = (\ell )$ is a unit term, then $\sigma$ has to be extended by $\overline{\ell }$ in order not to satisfy *T*. If several clauses or terms are unit under $\sigma$, QBCP nondeterministically picks one and extends the assignment accordingly. This is repeated until a constraint is empty or no unit constraints remain.

If QBCP does not lead to an empty constraint, the assignment $\sigma$ is extended by *branching*. That is, the solver chooses an unassigned variable *y* such that every variable *x* with $(x, y) \in D_{\varPhi }$ is assigned, and extends the assignment $\sigma$ by *y* or $\lnot y$.

The resulting assignment can be partitioned into so-called *decision levels*. The decision level of an assignment $\sigma$ is the number of literals in $\sigma$ that were assigned by branching. The decision level of a literal $\ell$ in $\sigma$ is the decision level of the prefix of $\sigma$ that ends with $\ell$. Note that each decision level greater than 0 can be associated with a unique variable assigned by branching.

Eventually, the assignment maintained by QCDCL must falsify a clause or satisfy a term. When this happens (we call this a *conflict*), the solver proceeds to *conflict analysis* to derive a learned constraint *C*. Initially, *C* is the falsified clause (satisfied term), called the *conflict clause (term)*. The solver finds the existential (universal) literal in *C* that was assigned last by QBCP, and the antecedent clause (term) *R* responsible for this assignment. A new constraint is derived by resolving *C* and *R* and applying universal (existential) reduction (again, relative to $D_{\varPhi }$). This is done repeatedly until the resulting constraint *C* is *asserting*. A clause (term) *S* is asserting if there is a unique existential (universal) literal $\ell \in S$ with maximum decision level (greater than zero) among literals in *S*, the corresponding decision variable is existential (universal), and every universal (existential) variable $y \in var (S)$ such that $(y, var (\ell )) \in D_{\varPhi }$ is assigned at a lower decision level (an asserting constraint becomes unit after backtracking). Once an asserting constraint has been found, it is added to the solver’s set of constraints. Finally, QCDCL *backtracks*, undoing variable assignments until reaching a decision level computed from the learned constraint.

Pseudocode for the main QCDCL loop is shown as Algorithm 1, and pseudocode for conflict analysis is shown as Algorithm 2. We now formally state and prove a correspondence between clauses learned by QCDCL and LDQ(D)-resolution.

### Proposition 2

Every clause learned by Algorithm 1 given an input PCNF $\varPhi$ can be derived from $\varPhi$ by LDQ(D)-resolution.

### Proof

We will show that each learned clause constructed during conflict analysis can be derived by LDQ(D)-resolution from input clauses or previously learned clauses. In addition, we prove an invariant saying that each such clause *C* can be reduced to the empty clause under the trail assignment restricted to variables up to and including the existential variable assigned last among those in *C*. Formally, if $\ell _1\dots \ell _k$ is the trail and $i = \max \{\,j \;{:}\;\overline{\ell _j} \in C, 1 \le j \le k \,\}$, then the restricted trail is $\ell _1\dots \ell _i$. If such an *i* does not exist—which can happen only if *C* contains no existential variable—the restricted trail is empty. Let $\tau$ denotes the assignment corresponding to the restricted trail. We want to show that $C[\tau ]$ simplifies to the empty clause by $\forall$-reduction. The proof is by induction on the number of resolution operations performed by Algorithm 2.

The base case with *C* being the conflict clause is trivial. For the inductive step, suppose that *C* can be reduced to the empty clause under the restricted trail assignment $\tau = \ell _1\dots \ell _i$. Thus $\overline{\ell _i} \in C$ is the existential literal falsified last among literals in *C* and conflict analysis would resolve *C* with the antecedent *R* of $\ell _i$ next. We have to show the resolvent satisfies the above invariant and that this resolution operation is permissible in LDQ(D)-resolution. Let $\tau ' = \ell _1\dots \ell _{i-1}$ denote the trail at the time when $\ell _i$ was propagated. Clause *R* is unit under $\tau '$, that is, $R[\tau ']$ reduces to $(\ell _i)$. In particular, we have $\tau '(\ell ) = 0$ for every existential literal $\ell \in R {\setminus } \{\ell _i\}$ and every universal literal $\ell \in R$ assigned by $\tau '$. Since $C[\tau ]$ reduces to the empty clause by assumption, we further must have $\tau (\ell ) = 0$ for every existential literal and every assigned universal literal of *C*. We conclude that $\tau '(\ell ) = 0$ for every existential literal and every assigned universal literal in the resolvent $C' = (C \cup R) {\setminus } \{\ell _i, \overline{\ell _i}\}$. This property is clearly not affected by unassigning universal variables or unassigning existential variables not occurring in $C'$, so $C'$ reduces to the empty clause under its corresponding restricted trail assignment, proving that the invariant is preserved. Furthermore, it entails that any literal $\ell \in R$ such that $\overline{\ell } \in C$ must be universal and unassigned by $\tau '$. Since $R[\tau ']$ can be reduced to $(\ell _i)$ by $\forall$-reduction, we must have $( var (\ell ), var (\ell _i)) \notin D_{\varPhi }$ for each such literal $\ell$, so $C'$ can be derived from *C* and *R* in LDQ(D)-resolution. $\square$


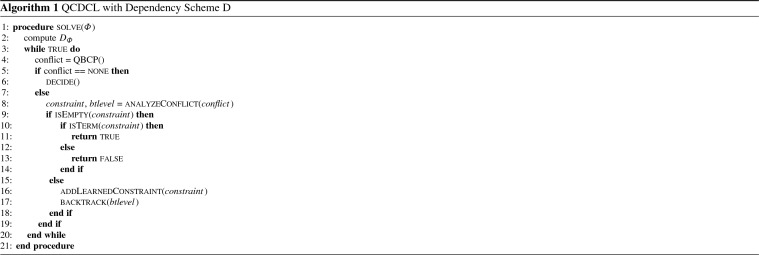
 
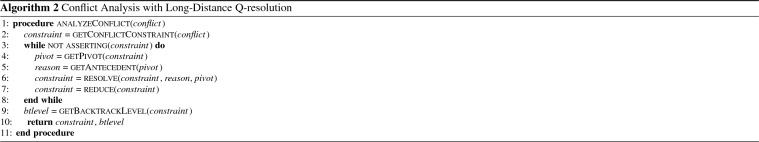


As in the case of QCDCL without dependency schemes [19, 21] an analogue of this result can be proved for learned terms and a dual proof system (“Q-consensus”) that operates on terms instead of clauses.

The proof of Proposition 2 uses the fact that two clauses $C_1 \vee u \vee e$ and $C_2 \vee \lnot u \vee \lnot e$ can be resolved on variable *e* even if $u < e$ as long as $(u, e) \notin D_{\varPhi }$. The following example illustrates that this generalization of the resolution rule is necessary for LDQ(D)-resolution to trace QCDCL with dependency schemes and long-distance Q-resolution.

### Example 1

Consider the formula $\varPhi = \exists z_0 \exists z_1 \forall x \exists y \exists z_2 \exists z_3 \exists a \exists b. \varphi \wedge \psi$, where

$$\begin{aligned} \varphi = \underbrace{(\overline{z_1} \vee x \vee z_2 \vee \overline{a})}_{C_1} \wedge \underbrace{(z_1 \vee y \vee z_2)}_{C_2} \wedge \underbrace{(\overline{x} \vee \overline{y} \vee z_3 \vee \overline{b})}_{C_3} \wedge \underbrace{(\overline{z_2} \vee z_0)}_{C_4} \wedge \underbrace{(\overline{z_2} \vee \overline{z_0})}_{C_5} \wedge \underbrace{(\overline{y} \vee \overline{z_3})}_{C_6}, \end{aligned}$$

and $\psi$ consists of auxiliary clauses

$$\begin{aligned} \psi = (a) \wedge (b) \wedge (\overline{x} \vee a) \wedge (\overline{x} \vee b). \end{aligned}$$

The clauses in $\psi$ are there simply to enforce that *a* and *b* are set to true and (in conjunction with $\varphi$) that $(x, a), (x, b) \in \text { D }^{\text { rrs}}_{\varPhi }$. It is straightforward to check that the set of dependencies computed by the reflexive resolution-path dependency scheme is

$$\begin{aligned} \text { D }^{\text { rrs}}_{\varPhi } = \{ (z_0, x), (z_1, x), (x, a), (x, b) \}. \end{aligned}$$

That is, the dependency scheme identifies the syntactic dependencies $(x, y), (x, z_2)$, and $(x, z_3)$ as spurious.

We now construct a possible trace of QCDCL on $\varPhi$ with $\text { D }^{\text { rrs}}$ and learning based on long-distance Q-resolution. At decision level 0 the unit clauses in $\psi$ are propagated, setting $a = 1$ and $b = 1$. This does not lead to further propagation and QCDCL proceeds with the decision $y \overset{d}{=} 0$. Note that *y* can be assigned before *x* because $(x, y) \notin \text { D }^{\text { rrs}}_{\varPhi }$. This assignment does not lead to any literals being propagated, so the algorithm makes another decision $z_2 \overset{d}{=} 0$. Now clause $C_2$ simplifies to the unit clause $(z_1)$ and $z_1 = 1$ is propagated. Clause $C_1$ only contains *x* under the resulting assignment and we have a conflict. Conflict analysis first resolves clauses $C_1$ and $C_2$ to obtain the clause $C_{12} = (x \vee y \vee z_2 \vee \lnot a)$. Variable *a* depends on *x*, so $\forall$-reduction cannot be applied. Since $z_2$ is the only variable from the second decision level in clause $C_{12}$ and $z_2$ does not depend on *x*, $C_{12}$ is asserting and the clause is learned by QCDCL. Backtracking undoes the decision involving $z_2$ and propagates $z_2 = 1$ instead. As a result, $C_4$ simplifies to $(z_0)$, unit propagation assigns $z_0 = 1$, and clause $C_5$ is falsified. Conflict analysis resolves $C_4$ and $C_5$ to derive the learned (unit) clause $C_{45} = (\overline{z_2})$, which causes QCDCL to backtrack to decision level 0 and propagate $z_2 = 0$. Now clause $C_{12}$ simplifies to $(x \vee y)$. Since *y* is independent of *x* we can apply $\forall$-reduction to obtain the unit clause (*y*) which propagates the assignment $y = 1$. Clause $C_6$ in turn becomes unit and propagates $z_3 = 0$. As a result, clause $C_3$ simplifies to $(\overline{x})$ and reduces to the empty clause by $\forall$-reduction. Conflict analysis resolves $C_3$ and $C_6$ so as to obtain the clause $(\overline{x} \vee \overline{y} \vee \overline{b})$. Variable *b* depends on *x*, so $\forall$-reduction cannot be applied. Next, clause $(\overline{x} \vee \overline{y} \vee \overline{b})$ is resolved with $C_{12}$ to derive $(x \vee \overline{x} \vee z_2 \vee \overline{a} \vee \overline{b})$. Note that this resolution step is permissible since $(x, y) \notin \text { D }^{\text { rrs}}_{\varPhi }$. Further resolution steps involving unit clauses yield the clause $(x \vee \overline{x})$, which can be reduced to the empty clause, so that QCDCL terminates with return value FALSE.

## Soundness of and Strategy Extraction for LDQ(D$^\text {rrs}$)

### Polynomial-Time Strategy Extraction from LDQ(D)-Refutations

A PCNF formula can be associated with an evaluation game played between an existential and a universal player. These players take turns assigning quantifier blocks in the order of the prefix. The existential player wins if the matrix evaluates to 1 under the resulting variable assignment, while the universal player wins if the matrix evaluates to 0. One can show that the formula is true (false) if and only if the existential (universal) player has a winning strategy in this game, and this winning strategy is a (counter)model.

Goultiaeva et al. [24] proved that a Q-resolution refutation can be used to compute winning moves for the universal player in the evaluation game. The idea is that universal maintains a “restriction” of the refutation by the assignment constructed in the evaluation game, which is a refutation of the restricted formula.

For assignments made by the existential player, the universal player only needs to consider each instance of resolution whose pivot variable is assigned: one of the premises is not satisfied and can be used to (re)construct a refutation.

When it is universal’s turn, the quantifier block for which she needs to pick an assignment is leftmost in the restricted formula. This means that $\forall$-reduction of these variables is blocked by any of the remaining existential variables and can only be applied to a purely universal clause. In a Q-resolution refutation, these variables must therefore be reduced at the very end, and because Q-resolution does not permit tautological clauses, only one polarity of each universal variable from the leftmost block can appear in a refutation. It follows that universal can maintain a Q-resolution refutation by assigning variables from the leftmost block in such a way as to map the associated literals to 0, effectively deleting them from the remaining Q-resolution refutation.

In this manner, the universal player can maintain a refutation until the end of the game, when all variables have been assigned. At that point, a refutation consists only of the empty clause, which means that the assignment chosen by the two players falsifies a clause of the original matrix and universal has won the game.

Egly et al.  [19] observed that this argument goes through even in the case of long-distance Q-resolution, since a clause containing both *u* and $\lnot u$ for a universal variable *u* can only be derived by resolving on an existential variable to the left of *u*, but no such existential variable exists if *u* is from the leftmost block.

In this section, we will prove that this argument can be generalized to LDQ(D$^\text {rrs}$)-refutations. We illustrate this correspondence with an example:

#### Example 2

Consider the PCNF formula

$$\begin{aligned} \varPhi =\;&\exists x\,\forall u\,\exists e, y\quad (x\vee u\vee \overline{y})\wedge (\overline{x}\vee \overline{u}\vee \overline{y})\wedge (x\vee y)\wedge (\overline{x}\vee e)\wedge (\overline{u}\vee y)\wedge (\overline{y}\vee e) \end{aligned}$$

**Fig. 2:**
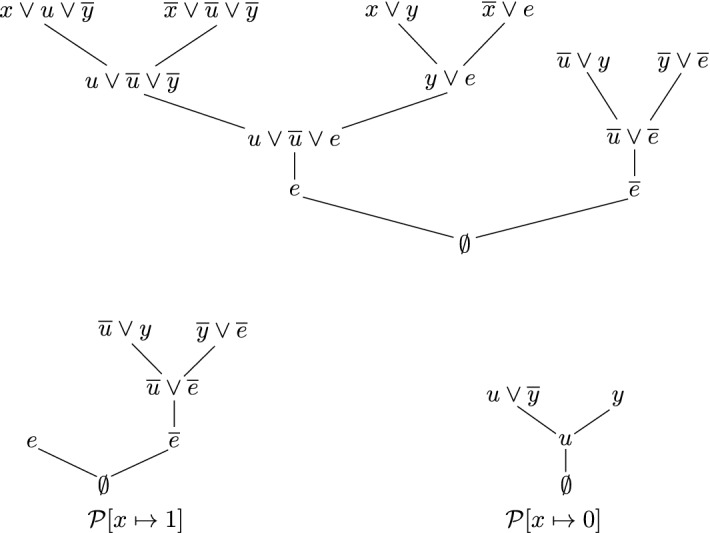
An LDQ(D$^\text {rrs}$)-refutation of the formula $\varPhi$ from Example 2 (above) and two restrictions (below)

Figure 2 shows an LDQ(D$^\text {rrs}$)-refutation of $\varPhi$. The only universal variable is *u*, so a strategy for the universal player in the evaluation game associated with $\varPhi$ has to determine an assignment to *u* given an assignment to *x*, the only (existential) variable preceding *u*. The figure illustrates how to compute the assignment to *u* for the two possible assignments $\tau : \{x\} \rightarrow \{0, 1\}$ from the restriction of the refutation by $\tau$. In both cases, only one polarity of *u* occurs in the restricted refutation and therefore it is easy for universal to determine the correct assignment. Notice that in one of the cases, a generalized $\forall$-reduction node remains present in the restriction—this shows that we cannot limit ourselves to looking at the final reduction step in the proof when looking for the variables to assign (as is the case with ordinary Q-resolution refutations, cf. [24]).

The key property that allows universal to maintain a refutation in the above example is that universal variables from the leftmost quantifier block may appear in at most one polarity. We now show that this property is in fact sufficient for soundness of LDQ(D) when combined with a natural monotonicity property of dependency schemes.

#### Definition 7

A dependency scheme D is *monotone* if $D_{\varPhi [\tau ]}\subseteq D_{\varPhi }$ for every PCNF formula $\varPhi$ and every assignment $\tau$ to a subset of $var (\varPhi )$. We say that D is *simple* if, for every PCNF formula $\varPhi =\forall X\mathcal {Q}.\varphi$, every LDQ(D)-derivation $\mathcal {P}$ from $\varPhi$, and every universal variable $u \in X$, *u* or $\overline{u}$ does not appear in $\mathcal {P}$. A dependency scheme D is *normal* if it is both monotone and simple.

As in the case of Q-resolution, universal’s move for a particular quantifier block can be computed from the assignment corresponding to the previous moves and the refutation in polynomial time. Since every polynomial-time algorithm can be implemented by a family of polynomially-sized circuits, and because these circuits can even be computed in polynomial time [1, p. 109], it follows that LDQ(D) admits polynomial-time strategy extraction when D is normal (in “Appendix Appendix:”, we present an explicit construction with a more specific bound on the runtime).

#### Theorem 1

Let *D* be a normal dependency scheme. There is a polynomial-time algorithm that, given a PCNF formula $\varPhi$ and an LDQ(D)-refutation of $\varPhi$, computes a countermodel of $\varPhi$.

As an application of this general result, we will prove that the reflexive resolution-path dependency scheme is normal in Sect. 5.2 below.

#### Theorem 2

$$\mathrm {\text { D }^{\text { rrs}}}$$ is normal.

#### Corollary 1

There is a polynomial-time algorithm that, given a PCNF formula $\varPhi$ and an LDQ(D$^\text {rrs}$)-refutation of $\varPhi$, computes a countermodel of $\varPhi$.

This result immediately carries over to the less general standard dependency scheme.

#### Corollary 2

There is a polynomial-time algorithm that, given a PCNF formula $\varPhi$ and an LDQ(D$^\text {std}$)-refutation of $\varPhi$, computes a countermodel of $\varPhi$.

In combination with Proposition 1, these results imply soundness of both proof systems.

#### Corollary 3

The systems LDQ(D$^\text {std}$) and LDQ(D$^\text {rrs}$) are sound.

### The Reflexive Resolution-Path Dependency Scheme is Normal

In order to prove Theorem 2 and show that $\text { D }^{\text { rrs}}$ is normal, we will need some insight into the relationship between resolution paths and LDQ(D$^\text {rrs}$)-derivations. For a formula $\varPhi$ and a universal literal *u*, we will denote by $T_u(\varPhi )$ the set of existential literals *e* such that $u < e$ and such that *e* is reachable from *u* by a resolution path via existential variables to the right of *u* in $\varPhi$.

#### Lemma 1

Let $\varPhi = \forall X \mathcal {Q}.\varphi$ be a PCNF formula and let *u* be a universal literal with $var (u) \in X$. Let $C_1, C_2\in \varphi$ be clauses such that for some existential literal *x*, $x\in C_1$ and $\overline{x}\in C_2$, and let $C=C_1\cup C_2{\setminus }\{x,\overline{x}\}$. Then $T_u(\varPhi )=T_u(\mathcal {Q}.\varphi \cup \{C\} )$.

#### Proof

Let $\varPhi '= \mathcal {Q}.\varphi \cup \{C\}$. Of course, by adding clauses to a formula, we preserve all existing resolution paths, so $T_u(\varPhi )\subseteq T_u(\varPhi ')$. We will prove that the opposite inclusion holds as well. Let $e\in T_u(\varPhi ')$ and let $\pi$ be a resolution path in $\varPhi '$ certifying this. If $\pi$ is also a resolution path in $\varPhi$, we are done. If it is not, it must be because it performs a *C-transition*, namely it contains two consecutive literals $l_1, l_2$ such that $var(l_1)\ne var(l_2)$, $l_1,l_2\in C$, but $\{l_1,l_2\}\not \subseteq C_1$ and $\{l_1,l_2\}\not \subseteq C_2$. In this case, without loss of generality, we have $l_1\in C_1$ and $l_2\in C_2$. Let $\pi _1$ be the prefix of $\pi$ up to and including $l_1$ and $\pi _2$ be the suffix of $\pi$ starting with $l_2$. Let $\pi '$ be the concatenation of $\pi _1$, *x*, $\overline{x}$, and $\pi _2$. It is clearly a valid resolution path and it uses one fewer *C*-transitions than $\pi$. Iterating this process, we can remove all *C*-transitions from $\pi$ to obtain a resolution path in $\varPhi$. The resulting resolution path has the same endpoints and therefore certifies that $e\in T_u(\varPhi )$. $\square$

The previous lemma implies that when considering reachability from an outermost universal literal in a formula $\varPhi$, we can use clauses derived from $\varPhi$ by LDQ(D$^\text {rrs}$)-resolution as well. Indeed, adding clauses produced by the resolution rule does not change the set of reachable literals by Lemma 1, and adding clauses produced by universal reduction clearly does not even create new resolution paths. Particularly, if two literals ever appear together in a derived clause, there is a resolution path between them. This is summarized by the following corollary.

#### Corollary 4

Let $\mathcal {P}$ be an LDQ(D$^\text {rrs}$)-derivation from a PCNF formula $\varPhi = \forall X \mathcal {Q}.\varphi$ and let $u\in X, u\in Cl(\mathcal {P})$. Then for all existential literals $e\in Cl(\mathcal {P})$, there is a resolution path from *u* to *e* in $\varPhi$.

As a first step towards proving Theorem 2, we will prove that both polarities of an outermost universal literal cannot appear together in a single clause of a derivation.

#### Lemma 2

Let $\mathcal {P}$ be an LDQ(D$^\text {rrs}$)-derivation from a PCNF formula $\varPhi = \forall X \mathcal {Q}.\varphi$ and let $u\in X$. Then $u\notin Cl(\mathcal {P})$ or $\lnot u\notin Cl(\mathcal {P})$.

#### Proof

Towards a contradiction, suppose $u,\lnot u\in Cl(\mathcal {P})$. Since input clauses do not contain both polarities of any literal, there must be a resolution step inside the derivation, which merges *u* and $\lnot u$ into one clause. Let $\mathcal {P}'=\mathcal {P}_1\odot _x \mathcal {P}_2$ be such a step. Then, without loss of generality, $x,u\in Cl(\mathcal {P}_1)$ and $\lnot x,\lnot u\in Cl(\mathcal {P}_2)$ and by Corollary 4, there is a resolution path from *u* to *x* and from $\lnot u$ to $\lnot x$, i.e. $(u,x)\in \text { D }^{\text { rrs}}_\varPhi$. However, if *x* depends on *u*, opposite polarities of *u* cannot be merged in a resolution step with the pivot *x*, a contradiction. $\square$

We will further assume that the derivation considered in the proof of Theorem 2 is in the normal form given by the following lemma.

#### Lemma 3

Let $\mathcal {P}$ be an LDQ(D$^\text {rrs}$)-derivation from a PCNF formula $\varPhi = \forall X \mathcal {Q}.\varphi$, let $u \in X$ be a universal variable such that both *u* and $\lnot u$ appear in $\mathcal {P}$, and let $Y = var _{\exists }(\varPhi )$. There exists an LDQ(D$^\text {rrs}$)-derivation $\mathcal {P}'$ from a PCNF formula $\varPhi ' = \forall u \exists Y.\varphi '$ such that $\mathcal {P}'$ contains both *u* and $\lnot u$ but only $\forall$-reduction steps with respect to one polarity of *u*.

#### Proof

Let $\varphi '$ be the result of removing all universal variables except *u* from $\varphi$. Removing universal variables does not introduce new resolution paths, so $\text { D }^{\text { rrs}}_{\varPhi '} \subseteq \text { D }^{\text { rrs}}_{\varPhi }$. The derivation $\mathcal {P}'$ can be obtained from $\mathcal {P}$ in the following way. We first delete all occurrences of universal variables other than *u* from $\mathcal {P}$, along with $\forall$-reduction steps involving such variables. The result is an LDQ(D$^\text {rrs}$)-derivation from $\varPhi '$, and it still contains both *u* and $\lnot u$. Next, we choose a subderivation containing both *u* and $\lnot u$ such that none of its proper subderivations contains both literals. By Lemma 2, the conclusion *C* of this derivation can contain at most one of *u* and $\lnot u$. If $u \in C$ we omit all $\forall$-reduction steps involving *u*. Otherwise, we omit all $\forall$-reduction steps involving $\lnot u$. This yields the desired LDQ(D$^\text {rrs}$)-derivation $\mathcal {P}'$ from $\varPhi '$. $\square$

**Fig. 3:**
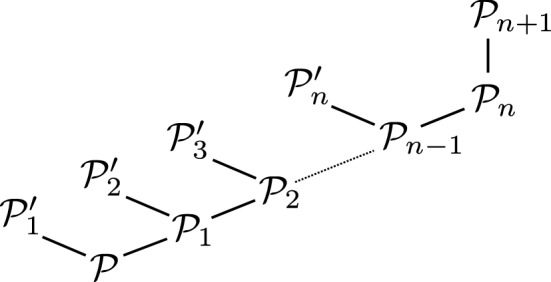
Shape of the derivation constructed in the proof of Theorem 2

Using Lemmas 2 and  3, we can proceed to finish the proof of Theorem 2.

#### Proof of Theorem 2

Towards a contradiction, consider an LDQ(D$^\text {rrs}$)-derivation from a formula $\varPhi = \forall X \mathcal {Q}.\varphi$ and let $u\in X$ be such that both polarities of *u* occur in this derivation. Let $Y = var _{\exists }(\varPhi )$ and let $\mathcal {P}$ denote the simplified derivation given by Lemma 3. Assume without loss of generality that $\mathcal {P}$ is a tree (any derivation can be turned into a tree-like derivation by copying proof nodes), and that $\mathcal {P}$ does not contain $\forall$-reduction steps involving $\lnot u$. Since $\lnot u$ occurs in $\mathcal {P}$ but is not reduced, $\lnot u$ must occur in the conclusion of $\mathcal {P}$. Thus *u* cannot occur in the conclusion by Lemma 2. Since *u* is present in the derivation $\mathcal {P}$, this means there must be a reduction step on *u* somewhere in $\mathcal {P}$. As *u* is the only universal variable and we omitted all reduction steps on $\lnot u$, all reduction steps in $\mathcal {P}$ are on *u* and $\mathcal {P}$ must have the form depicted in Fig. 3, where $\mathcal {P}_n=\mathcal {P}_{n+1}-u$ is a lowermost reduction step on *u* and the subsequent resolutions are on pivots $x_n,\ldots , x_1$. Let $C_0=Cl(\mathcal {P}), C_i=Cl(\mathcal {P}_i), C_i'=Cl(\mathcal {P}_i')$. The clauses $C_1',\ldots ,C_n',C_{n+1}$ are derived by LDQ(D$^\text {rrs}$)-resolution and by Lemma 1 we know that we can use them to show resolution-path connections as if they were input clauses. By the transformations we considered we know that starting from an arbitrary LDQ(D$^\text {rrs}$)-derivation we can obtain a valid LDQ(D$^\text {rrs}$)-derivation in this form, so any contradiction we derive from here means a contradiction with the assumption that an LDQ(D$^\text {rrs}$)-derivation contains both polarities of a universal variable from the outermost block, thus proving Theorem 2. With that, we are ready to finish the proof.

We will prove that there is a resolution path from $\lnot u$ to *u* going through an existential literal in $C_{n+1}$, which is in contradiction with the soundness of reduction of *u* from $C_{n+1}$. Let us consider *open* resolution paths, i.e. resolution paths without their final literal. If an open resolution path ends in a literal $\ell$ of clause *C*, we say that the path *leads* to the clause *C*. By induction on *n*, we will prove that there is an open resolution path from $\lnot u$ which leads to the clause $C_n$. If $n=1$, we have the path $\lnot u, \lnot x_1, x_1$. For $n>1$, let $\pi$ be the open path leading to $C_{n-1}$ and let $\ell$ be its last literal. Then either $\ell \in C_n$, in which case we have an open path leading to $C_n$, or $\ell \in C_n'$, in which case we have the open path $\pi , \lnot x_n, x_n$ leading to $C_n$. An open path that leads to $C_n$ also leads to $C_{n+1}$, because those two clauses only differ in the presence of *u* and therefore can be closed by the literal *u* to obtain the required resolution path. $\square$

## Experiments

To gauge the potential of clause learning based on LDQ(D$^\text {std}$), we ran experiments with the search-based solver DepQBF^7^ in version 5.0. By default, DepQBF supports proof generation only in combination with the trivial dependency scheme—in that case, it generates Q-resolution or long-distance Q-resolution proofs (depending on whether long-distance resolution is enabled). However, by uncommenting a few lines in the source code, proof generation can also be enabled with the standard dependency scheme, and this option can even be combined with long-distance resolution. This leads to the solver generating Q(D$^\text {std}$)-resolution or LDQ(D$^\text {std}$)-resolution proofs (see Sect. 4).

We compared the performance of DepQBF in four configurations,^8^ each using a different proof system for constraint learning:

1. Long-distance Q-resolution with $\forall /\exists$-reduction according to D$^\text {std}$ (LDQD).
2. Long-distance Q-resolution with ordinary $\forall /\exists$-reduction (LDQ).
3. Q-resolution with $\forall /\exists$-reduction according to D$^\text {std}$ (QD).
4. Ordinary Q-resolution (Q).

These experiments were performed on a cluster with Intel Xeon E5649 processors at 2.53 GHz running 64-bit Linux. We set time and memory limits of 900 s and 4 GB, respectively. Instances were taken from two tracks of the QBF Gallery 2014: the *applications* track consisting of 6 instance families and a total of 735 formulas, and the *QBFLib* track consisting of 276 formulas.

For our first set of experiments, we disabled dynamic QBCE (Quantified Blocked Clause Elimination), a technique introduced with version 5.0 of DepQBF [32]. We further used bloqqer^9^ (version 037) with default settings as a preprocessor. Since LDQ(D$^\text {std}$) generalizes both long-distance Q-resolution and Q(D$^\text {std}$)-resolution, we were expecting a performance increase with LDQ(D$^\text {std}$)-learning compared to learning based on the other proof systems. However, all four configurations showed virtually identical performance on both the application and QBFlib benchmark sets in terms of total runtime and instances solved within the time limit (see Table 1).

**Table 1:** Solved instances, solved true instances, solved false instances, and total runtime in seconds (including timeouts) with preprocessing (but without QBCE)

Configuration	Solved	True	False	Time
Application track				
LDQD	377	186	191	343,455
LDQ	377	186	191	345,459
QD	377	183	194	343,928
Q	376	182	194	345,914
QBFLib track				
LDQD	130	69	61	140,743
LDQ	131	69	62	141,646
QD	129	67	62	140,975
Q	127	65	62	142,679

**Fig. 4:**
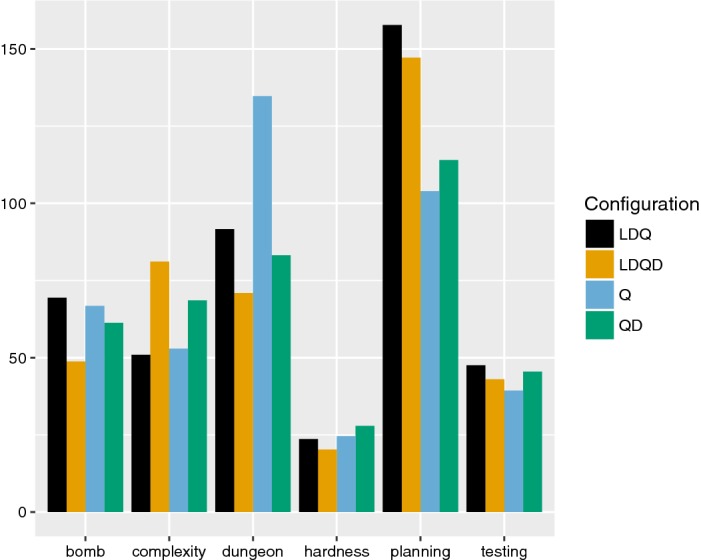
Average runtime in seconds (y-axis) for instances from the application track for each instance family (x-axis), by solver configuration (with preprocessing, but without dynamic QBCE). Here, we only considered instances that were solved by at least one configuration

To get a more detailed picture, we broke down the results for the application track by instance family, limiting ourselves to instances that were solved by at least one configuration. The barplot in Fig. 4 shows that there are considerable differences in performance between solver configurations for individual instances families, with each solver configuration being outperformed by another configuration on at least one family.

**Fig. 5:**
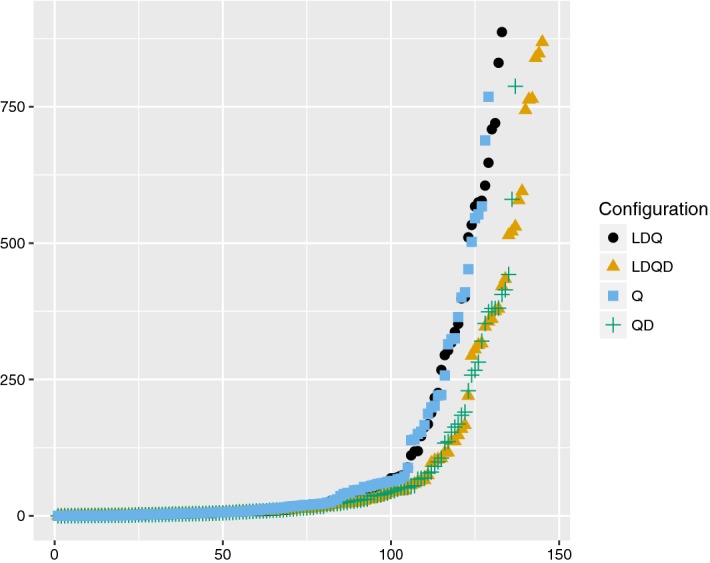
Solved instances from the QBFLib track (x-axis) sorted by runtime (y-axis), by solver configuration (with preprocessing and dynamic QBCE)

For our second set of experiments, we turned on dynamic QBCE. This led to a significant performance increase both in terms of number of instances solved within the time limit and total runtime for both benchmark sets, a result that is consistent with the findings in [32]. However, as far as the performance of LDQ(D$^\text {std}$)-learning is concerned, the application and QBFlib tracks differed significantly for this experiment. While LDQ(D$^\text {std}$)-learning fared *worst* among the configurations both with respect to instances solved and total runtime on the application track, it was the *best* configuration for the QBFlib track in both respects (see Table 2). Figure 5 shows that using the standard dependency scheme was beneficial both with and without long-distance resolution for the QBFlib instances.

**Table 2:** Results with preprocessing and dynamic QBCE

Configuration	Solved	True	False	Time
Application track				
LDQD	385	195	190	339,143
LDQ	388	201	187	336,739
QD	392	201	191	334,965
Q	389	198	191	337,141
QBFLib track				
LDQD	145	75	70	132,567
LDQ	133	64	69	141,682
QD	137	70	67	134,150
Q	129	62	67	142,399

For our final set of experiments, we left dynamic QBCE enabled but *disabled* preprocessing for the application track, as this was shown to lead to a performance *increase* in the case of learning with ordinary Q-resolution [32]. Indeed, this resulted in a performance increase across the board (see Table 3). Moreover, LDQ(D$^\text {std}$)-learning was the best configuration in terms of instances solved (on par with Q(D$^\text {std}$)-resolution) as well as in terms of overall runtime. Moreover, LDQ(D$^\text {std}$)-learning was the best configuration in terms of instances solved (on par with Q(D$^\text {std}$)-resolution) as well as in terms of overall runtime.

**Table 3:** Results for the application track with QBCE (but without preprocessing)

Configuration	Solved	True	False	Time
LDQD	440	223	217	287,012
LDQ	435	223	212	291,574
QD	440	225	215	291,661
Q	437	221	216	337,141

## Related Work

QCDCL with learning based on long-distance Q-resolution was first described by Zhang and Malik [45]. They presented an argument for the soundness of using tautological clauses (respectively, contradictory terms) within their algorithm but did not study long-distance Q-resolution as a proof system. Lacking a sound theoretical foundation, the use of tautological clauses in QCDCL was abandoned in favour of more complicated methods for constraint learning that avoid their generation [20, 21, 33].

Interest in long-distance Q-resolution was renewed when Balabanov and Jiang [2] introduced the proof system we presented in Sect. 3 (restricted to the trivial dependency scheme) and proved its soundness. Egly et al.  [19] showed that a family of formulas known to be hard for Q-resolution [28] admits short long-distance Q-resolution proofs. They also demonstrated that QCDCL with learning based on long-distance resolution generates long-distance Q-resolution proofs and presented a new version of DepQBF that implements this algorithm. Finally, they showed that a long-distance Q-resolution proof can be interpreted as a winning strategy in the evaluation game associated with a QBF, generalizing a result by Goultiaeva et al. [24]. While these results established a solid theoretical framework for the use of long-distance Q-resolution within QCDCL they did not provide an intuitive account of the semantics of individual tautological clauses. Such an account was subsequently presented by Balabanov et al. [3], who showed that tautological literals $u, \lnot u \in C$ can be interpreted as proxies for “phase functions” that determine whether a variable or its negation is present in clause *C* based on the values assigned to pivot variables appearing in the derivation of *C*. The authors used this interpretation to generalize the linear-time strategy extraction algorithm of Balabanov and Jiang [2] to long-distance Q-resolution proofs.

Recently and independently of this work, Beyersdorff and Blinkhorn investigated the soundness of Q-resolution proof systems parameterized by dependency schemes [6]. They define a property of dependency schemes D—*full exhibition*—which ensures that a certain version of long-distance Q(D)-resolution is sound, and show that the reflexive resolution-path dependency scheme has that property.

In a nutshell, a dependency scheme D is fully exhibited if every true QBF $\varPhi$ has a model $\{ f_e \}_{e \in var _{\exists }(\varPhi )}$ such that $f_e$ may only depend on a universal variable *u* if $(u, e) \in D_{\varPhi }$ (such models have elsewhere been referred to as *D-models* [40]). It is fairly straightforward to show that Q(D)-resolution is sound if D has this property, but generalizing this result to proof systems with long-distance resolution presents a challenge. Beyersdorff and Blinkhorn show that full exhibition is sufficient for soundness of a restricted version of LDQ(D)-resolution, where complementary universal literals that are “merged” by resolution must be annotated with the (existential) pivot variable, and universal reduction can be applied only if every existential variable occurring in the premise or the annotation of a universal variable is independent of the universal variable to be reduced. However, it is uncertain whether proofs generated by DepQBF with LDQ(D)-learning satisfy this additional restriction.

How full exhibition relates to our normality property is not entirely clear. Beyersdorff and Blinkhorn prove that full exhibition is *not* sufficient for soundness of LDQ(D)-resolution as defined here. In combination with Theorem 1, this shows that dependency schemes that are fully exhibited need not be normal. Whether there are normal dependency schemes that are not fully exhibited, on the other hand, remains open. Indeed, there is some evidence to the effect that normality entails full exhibition: consider a dependency scheme D that is not fully exhibited, and let $\varPhi = \forall u \mathcal {Q}.\varphi$ be a true QBF that does not have a D-model. Suppose *u* is the only universal variable of $\varPhi$. In this restricted case, the (non-)existence of a D-model can be expressed as a QBF $\varPsi$ by simply shifting existentials independent of *u* to the left. Because $\varPhi$ does not have a D-model, $\varPsi$ must be false and admit a Q-resolution refutation $\mathcal {P}$, which is also an LDQ(D)-refutation of $\varPhi$. Because $\varPhi$ is true, LDQ(D)-resolution must be unsound and so D cannot be normal by Theorem 1. Obviously, the assumption that *u* is the only universal variable of $\varPhi$ is very restrictive, but since we can suppose that D is monotone (recall that a dependency scheme is normal if it is both simple and monotone), there is hope that the argument for an arbitrary QBF can be reduced to this case by instantiating with a suitable variable assignment.

## Discussion

The results of Sects. 4 and  5 establish a partial soundness proof of QCDCL with learning based on LDQ(D$^\text {std}$): we now know that we can trust such a solver when it outputs “false”. To prove that “true” answers can be trusted as well, one has to show soundness of quantified term resolution (Q-consensus) when combined with the standard dependency scheme and long-distance resolution. The reason this does not follow from the results proved here is that they rely on a correspondence of Q-resolution derivations with dependency-inducing resolution paths that is not immediate for terms generated from an input PCNF: there is a correspondence of Q-consensus derivations with dual “resolution paths” that connect such terms, but these “resolution paths” do not induce resolution-path dependencies in the input PCNF.

The experiments in Sect. 6 indicate that we should not expect significant performance gains when switching from learning with Q(D$^\text {std}$)-resolution to LDQ(D$^\text {std}$). This is in spite of the fact that, from a purely theoretical perspective, LDQ(D$^\text {std}$) is a stronger proof system: a well-studied class of QBFs introduced by Kleine Büning, Karpinski, and Flögel requires exponentially-sized Q-resolution proofs [28] but admits short long-distance Q-resolution refutations [19], and since the standard dependency scheme does not offer any improvement over trivial dependencies on these formulas (see [12]) we obtain an exponential separation of LDQ(D$^\text {std}$)-resolution from Q(D$^\text {std}$)-resolution. From a practical point of view, the main benefit of using LDQ(D$^\text {std}$)-resolution over Q(D$^\text {std}$)-resolution is that conflict analysis is much simpler (cf. [19]). A learned constraint can be obtained from a conflict simply by resolving variables in the reverse order of their propagation (see Sect. 4). Methods that avoid the generation of tautological clauses (contradictory terms) during learning are significantly more involved [20, 21, 33].

We have shown that LDQ(D$^\text {rrs}$)-refutations allow for polynomial-time strategy extraction. In practice, the corresponding algorithm generates circuits that are frequently larger by an order of magnitude than the refutations provided as input. It is unclear whether this increase in size can be avoided by careful engineering alone or only by using a different approach. Faster (linear time) strategy extraction algorithms are known for “ordinary” Q-resolution and long-distance Q-resolution [2, 3]. Unfortunately, their underlying idea of setting universal variables so as to falsify the premise of some $\forall$-reduction step no longer works when dependency schemes enter the mix: generalized $\forall$-reduction may remove a universal variable *u* even in the presence (in the premise) of an existential variable *e* such that $u < e$ and the universal player can only be sure to falsify the premise if the *e*-literal is false, but she does not know the value of *e* at the time of assigning *u*. We believe that developing linear-time strategy extraction algorithms for Q(D)-resolution or LDQ(D)-resolution is going to require a better understanding of the power of these proof systems vis-à-vis Q-resolution and long-distance Q-resolution [12].

## Proof of Theorem 1

We begin by formally defining the “restriction” of an LDQ(D)-derivation by an assignment, which is a straightforward generalization of this operation for Q-resolution derivations [24].^10^ The result of restricting a derivation is either a derivation or the object $\top$, which can be interpreted as representing the tautological clause containing every literal. Accordingly, we stipulate that $\ell \in \top$ for every literal $\ell$.

### Definition 8

(*Restriction*) Let $\varPhi$ be a PCNF formula and let $\mathcal {P}$ be an LDQ(D)-derivation from $\varPhi$. Further, let $X \subseteq var (\varPhi )$ and let $\tau : X \rightarrow \{0, 1\}$ be a truth assignment. The *restriction of*$\mathcal {P}$*by*$\tau$, in symbols $\mathcal {P}[\tau ]$, is defined as follows.

1. If $\mathcal {P}$ is an input node there are two cases. If $Cl(\mathcal {P})[\tau ] = 1$ then $\mathcal {P}[\tau ] = \top$. Otherwise, $\mathcal {P}[\tau ]$ is the proof DAG consisting of a single node labeled with $Cl(\mathcal {P})[\tau ]$.
2. If $\mathcal {P}= \mathcal {P}_1 \odot _{\ell } \mathcal {P}_2$, that is, if $\mathcal {P}$ is a resolution node, we distinguish four cases:
  - If $$\ell \notin Cl(\mathcal {P}_1[\tau ])$$ then $$\mathcal {P}[\tau ] = \mathcal {P}_1[\tau ]$$.
  - If $\ell \in Cl(\mathcal {P}_1[\tau ])$ and $\overline{\ell } \notin Cl(\mathcal {P}_2[\tau ])$ then $\mathcal {P}[\tau ] = \mathcal {P}_2[\tau ]$.
  - If $\ell \in Cl(\mathcal {P}_1[\tau ])$, $\overline{\ell } \in Cl(\mathcal {P}_2[\tau ])$, and $\mathcal {P}_1[\tau ] = \top$ or $\mathcal {P}_2[\tau ] = \top$, we let $\mathcal {P}[\tau ] = \top$.
  - If $\ell \in Cl(\mathcal {P}_1[\tau ])$, $\overline{\ell } \in Cl(\mathcal {P}_2[\tau ])$, $\mathcal {P}_1[\tau ] \ne \top$, and $\mathcal {P}_2[\tau ] \ne \top$, we define $\mathcal {P}[\tau ] = \mathcal {P}_1[\tau ] \odot _{\ell } \mathcal {P}_2[\tau ]$.
3. If $\mathcal {P}= \mathcal {P}' - u$, that is, if $\mathcal {P}$ is a $\forall$-reduction node, we distinguish three cases:
  - If $$\mathcal {P}'[\tau ] = \top $$ then $$\mathcal {P}[\tau ] = \top $$.
  - If $\mathcal {P}'[\tau ] \ne \top$ and $u \notin var (Cl(\mathcal {P}'[\tau ]))$ then $\mathcal {P}[\tau ] = \mathcal {P}'[\tau ]$.
  - If $\mathcal {P}'[\tau ] \ne \top$ and $u \in var (Cl(\mathcal {P}'[\tau ]))$ then $\mathcal {P}[\tau ] = \mathcal {P}'[\tau ] - u$.

If D is a monotone dependency scheme, LDQ(D)-refutations are preserved under restriction by an existential assignment (cf. [24, Lemma 4]). This is stated in the following lemma, which can by proved by a straightforward induction on the structure of the LDQ(D)-derivation.

### Lemma 4

Let D be a monotone dependency scheme, let $\mathcal {P}$ be an LDQ(D)-derivation from a PCNF formula $\varPhi$, let $E \subseteq var _{\exists }(\varPhi )$, and let $\tau : E \rightarrow \{0, 1\}$ be an assignment. If $\mathcal {P}[\tau ] = \top$ then $Cl(\mathcal {P})[\tau ] = 1$. Otherwise, $\mathcal {P}[\tau ]$ is an LDQ(D)-derivation from $\varPhi [\tau ]$ such that $Cl(\mathcal {P}[\tau ])\subseteq Cl(\mathcal {P})[\tau ]$.

### Proof

The proof is by induction on the structure of $\mathcal {P}$.

1. If $\mathcal {P}$ is an input node then $\mathcal {P}[\tau ] = \top$ iff $Cl(\mathcal {P})[\tau ] = 1$ and $Cl(\mathcal {P}[\tau ]) = Cl(\mathcal {P})[\tau ]$ otherwise, so the statement holds trivially.
2. If $\mathcal {P}= \mathcal {P}_1 \odot _\ell P_2$ is a resolution node we distinguish four cases:
  - If $\ell \notin Cl(\mathcal {P}_1[\tau ])$, then $\mathcal {P}[\tau ] = \mathcal {P}_1[\tau ]$ and
    $$\begin{aligned} Cl(\mathcal {P}_1[\tau ]) = Cl(\mathcal {P}_1[\tau ]) {\setminus } \{\ell \} \subseteq Cl(\mathcal {P}_1)[\tau ] {\setminus } \{\ell \} \subseteq Cl(\mathcal {P})[\tau ], \end{aligned}$$
    where the first inclusion holds by induction hypothesis and the second inclusion follows from the definition of the resolution rule.
  - If $\ell \in Cl(\mathcal {P}_1[\tau ])$ and $\overline{\ell }\notin Cl(\mathcal {P}_2[\tau ])$ then $\mathcal {P}[\tau ] = \mathcal {P}_2[\tau ]$ and the statement follows via a symmetric argument.
  - If $\ell \in Cl(\mathcal {P}_1[\tau ])$, $\overline{\ell }\in Cl(\mathcal {P}_2[\tau ])$, and $\mathcal {P}_1[\tau ]=\top$ or $\mathcal {P}_2[\tau ]=\top$ then we have $\mathcal {P}[\tau ] = \top$. Assume without loss of generality that $\mathcal {P}_1[\tau ]=\top$. Then $Cl(\mathcal {P}_1)[\tau ]=1$ by induction hypothesis. Let $\ell ' \in Cl(\mathcal {P}_1)$ be a literal such that $\tau (\ell ') = 1$. We distinguish two cases. If $\ell \ne \ell '$ then $\ell ' \in Cl(\mathcal {P})$ and $Cl(\mathcal {P})[\tau ] = 1$. Otherwise, $\tau (\overline{\ell '}) = \tau (\overline{\ell }) = 0$, and we must have $\mathcal {P}_2[\tau ] = \top$ since $\overline{\ell } \in Cl(\mathcal {P}_2[\tau ])$. By induction hypothesis, there has to be another literal $\ell '' \ne \overline{\ell }$ such that $\ell '' \in Cl(\mathcal {P}_2)$ and $\tau (\ell '') = 1$. The literal $\ell ''$ is contained in $Cl(\mathcal {P})$ as well, so $Cl(\mathcal {P})[\tau ] = 1$.
  - If $\ell \in Cl(\mathcal {P}_1[\tau ])$, $\overline{\ell } \in Cl(\mathcal {P}_2[\tau ])$, $\mathcal {P}_1[\tau ]\ne \top$, and $\mathcal {P}_2[\tau ]\ne \top$, then $\mathcal {P}[\tau ] = \mathcal {P}_1[\tau ] \odot _{\ell } \mathcal {P}_2[\tau ]$ and $\mathcal {P}[\tau ] \ne \top$. By induction hypothesis, $\mathcal {P}_1[\tau ]$ is an LDQ(D)-derivation from $\varPhi [\tau ]$ such that $Cl(\mathcal {P}_1[\tau ]) \subseteq Cl(P_1)[\tau ]$, and $\mathcal {P}_2[\tau ]$ is an LDQ(D)-derivation from $\varPhi [\tau ]$ such that $Cl(\mathcal {P}_2[\tau ]) \subseteq Cl(P_2)[\tau ]$. Monotonicity of *D* ensures that after restriction, the resolution step is still sound and thus $\mathcal {P}[\tau ]$ is an LDQ(D)-derivation from $\varPhi [\tau ]$ as well and
    $$\begin{aligned} Cl(\mathcal {P}[\tau ])&=Cl(\mathcal {P}_1[\tau ]\odot _{\ell } \mathcal {P}_2[\tau ]) \\&=Cl(\mathcal {P}_1[\tau ])\cup Cl(\mathcal {P}_2[\tau ]){\setminus }\{\ell ,\overline{\ell }\} \\&\subseteq Cl(\mathcal {P}_1)[\tau ]\cup Cl(\mathcal {P}_2)[\tau ]{\setminus }\{\ell ,\overline{\ell }\}=Cl(\mathcal {P})[\tau ]. \end{aligned}$$
3. If $\mathcal {P}= \mathcal {P}' - u$ is a reduction node, we have to distinguish two cases:
  - If $\mathcal {P}'[\tau ]=\top$ then $\mathcal {P}[\tau ]=\top$ by definition. By induction hypothesis $Cl(\mathcal {P}')[\tau ]=1$ and since $\tau$ does not assign *u*, we get $Cl(\mathcal {P})[\tau ]=1$ as well.
  - If $\mathcal {P}[\tau ]\ne \top$ then $\mathcal {P}'[\tau ] \ne \top$ by definition of the restriction operation. By induction hypothesis, $\mathcal {P}'[\tau ]$ is an LDQ(D)-derivation from $\varPhi [\tau ]$ such that $Cl(\mathcal {P}'[\tau ]) \subseteq Cl(\mathcal {P}')[\tau ]$. If $u \notin var (Cl(\mathcal {P}'[\tau ]))$ then $\mathcal {P}[\tau ] = \mathcal {P}'[\tau ]$ and the statement holds. Otherwise, if $u \in var (Cl(\mathcal {P}'[\tau ]))$ then $\mathcal {P}[\tau ] = \mathcal {P}'[\tau ] - u$ and thus
    $$\begin{aligned} Cl(\mathcal {P}[\tau ])&= Cl(\mathcal {P}'[\tau ]) {\setminus } \{u, \lnot u\}\\&\subseteq Cl(\mathcal {P}')[\tau ] {\setminus } \{u, \lnot u\} = (Cl(\mathcal {P}') {\setminus } \{u, \lnot u\})[\tau ]= Cl(\mathcal {P})[\tau ], \end{aligned}$$
    where the last but one equality holds because $\tau$ does not assign *u*. To see that $\mathcal {P}[\tau ] = \mathcal {P}'[\tau ] - u$ is a valid $\forall$-reduction node, note that $Cl(\mathcal {P}'[\tau ]) \subseteq Cl(\mathcal {P}')$ by induction hypothesis and observe that $D_{\varPhi [\tau ]} \subseteq D_{\varPhi }$.$\square$

Above, we argued that the universal player can use an LDQ(D)-refutation for a normal dependency scheme D in order to compute winning moves in the evaluation game associated with a PCNF formula and that this can be used to compute a countermodel of the formula in polynomial time. We now prove this directly, by showing how to construct a circuit implementing a countermodel from an LDQ(D)-refutation.

We begin by describing auxiliary circuits simulating the restriction operation. Let $\varPhi = Q_1X_1 \dots Q_k X_k.\varphi$ be a PCNF formula and let $\mathcal {P}$ be a refutation of $\varPhi$. For each quantifier block $X_i$, each subderivation ${\mathcal {S}}$ of $\mathcal {P}$, and each literal $\ell$, we will construct circuits $\textsc {top}^i_{\mathcal {S}}$ and $\textsc {contains}^i_{{\mathcal {S}}, \ell }$ with inputs from $X = \bigcup _{j < i}\,X_j$ such that, for every assignment $\sigma : X \rightarrow \{0, 1\}$,

1 $$\begin{aligned} \textsc {top}^i_{\mathcal {S}}[\sigma ] = 1&\Longleftrightarrow \mathcal {S}[\sigma ] = \top \end{aligned}$$

2 $$\begin{aligned} \textsc {contains}^i_{\mathcal {S}, \ell }[\sigma ] = 1&\Longleftrightarrow \ell \in Cl(\mathcal {S}[\sigma ]) \end{aligned}$$

We first describe our construction and then prove that it satisfies the above properties in Lemma 5. Let ${\mathcal {S}}$ be an input node. We let

$$\begin{aligned} \textsc {top}^1_{\mathcal {S}} := \bigvee \left( Cl(\mathcal {S}) \cap (X_1 \cup \overline{X_1})\right) , \end{aligned}$$

and define $\textsc {top}^i_{\mathcal {S}}$ for $1 < i \le k$ as

$$\begin{aligned} \textsc {top}^i_{\mathcal {S}}&:= \textsc {top}^{i-1}_{\mathcal {S}} \vee \bigvee \left( Cl(\mathcal {S}) \cap (X_i \cup \overline{X_i}) \right) . \end{aligned}$$

Moreover, for $1 \le i \le k$ we define $\textsc {contains}^i_{\mathcal {S}, \ell }$ as

$$\begin{aligned} \textsc {contains}^i_{\mathcal {S},{\ell }}&= {\left\{ \begin{array}{ll} 1 &{}\text {if }\ell \in Cl(\mathcal {S}) {\setminus } (X \cup \overline{X}),\\ \textsc {top}^i_{\mathcal {S}} &{}\text {otherwise.} \end{array}\right. } \end{aligned}$$

For non-input nodes, we proceed as follows. If ${\mathcal {S}} = {\mathcal {S}}_1 \odot _q {\mathcal {S}}_2$, we define $\textsc {top}^i_{\mathcal {S}}$ as

$$\begin{aligned} \textsc {top}^i_{\mathcal {S}} = (\textsc {contains}^i_{{\mathcal {S}}_1, q} \wedge \textsc {top}^i_{\mathcal {S}_2}) \vee (\textsc {contains}^i_{\mathcal {S}_2, \overline{q}} \wedge \textsc {top}^i_{\mathcal {S}_1}), \end{aligned}$$

and if ${\mathcal {S}} = {\mathcal {S}}' - u$, we let

$$\begin{aligned} \textsc {top}^i_{\mathcal {S}}&:= \textsc {top}^i_{\mathcal {S}'}. \end{aligned}$$

For the $\textsc {contains}^i_{\mathcal {S}, \ell }$ circuit, we distinguish two cases. Let $\ell$ be a literal and ${\mathcal {S}}$ a derivation. If $\ell \notin Cl(\mathcal {S})$ we simply let

$$\begin{aligned} \textsc {contains}^i_{{\mathcal {S}}, \ell } := \textsc {top}^i_{\mathcal {S}}. \end{aligned}$$

Otherwise, if $\ell \in Cl(\mathcal {S})$, we have to consider two cases. First, if ${\mathcal {S}} = {\mathcal {S}}_1 \odot _q {\mathcal {S}}_2$, we let

$$\begin{aligned} \textsc {contains}^i_{\mathcal {S}, \ell } =&\textsc {top}^i_{\mathcal {S}}\, \vee \\&(\lnot \textsc {contains}^i_{{\mathcal {S}}_1, q} \wedge \textsc {contains}^i_{\mathcal {S}_1, \ell })\, \vee \\&(\textsc {contains}^i_{\mathcal {S}_1, q} \wedge \lnot \textsc {contains}^i_{\mathcal {S}_2, \overline{q}} \wedge \textsc {contains}^i_{\mathcal {S}_2, \ell }) \, \vee \\&(\textsc {contains}^i_{{\mathcal {S}}_1, q} \wedge \textsc {contains}^i_{\mathcal {S}_2, \overline{q}} \wedge (\textsc {contains}^i_{\mathcal {S}_1, \ell } \vee \textsc {contains}^i_{\mathcal {S}_2, \ell })). \end{aligned}$$

Second, if ${\mathcal {S}} = {\mathcal {S}}' - u$, then

$$\begin{aligned} \textsc {contains}^i_{\mathcal {S}, \ell } := \textsc {contains}^i_{\mathcal {S}', \ell }. \end{aligned}$$

To implement the winning strategy for universal sketched above, we further construct circuits $\textsc {polarity}_{\mathcal {S}, u}$ for each node ${\mathcal {S}}$ of $\mathcal {P}$ and each universal variable $u \in var _{\forall }(\varPhi )$, such that, for each assignment $\tau : L_{\varPhi }(u) \rightarrow \{0, 1\}$,

3 $$\begin{aligned} \textsc {polarity}_{\mathcal {S}, u}[\tau ] = 1 \Longleftrightarrow { u} occurs in \mathcal {S}[\tau ] . \end{aligned}$$

Let $u \in X_i$ be a universal variable from the *i*th quantifier block. If ${\mathcal {S}}$ is an input node, we simply define

$$\begin{aligned} \textsc {polarity}_{{\mathcal {S}}, u} := \textsc {contains}^i_{\mathcal {S}, u}, \end{aligned}$$

and if ${\mathcal {S}} = {\mathcal {S}}' - u$ is a $\forall$-reduction node, we let

$$\begin{aligned} \textsc {polarity}_{\mathcal {S}, u} := \textsc {polarity}_{\mathcal {S}', u}. \end{aligned}$$

If $${\mathcal {S}} = {\mathcal {S}}_1 \odot _q {\mathcal {S}}_2$$, then

$$\begin{aligned} \textsc {polarity}_{\mathcal {S}, u} :=&(\lnot \textsc {contains}^i_{\mathcal {S}_1, q} \wedge \textsc {polarity}_{\mathcal {S}_1, u})\, \vee \\&(\textsc {contains}^i_{\mathcal {S}_1, q} \wedge \lnot \textsc {contains}^i_{\mathcal {S}_2, \overline{q}} \wedge \textsc {polarity}_{\mathcal {S}_2, u}) \, \vee \\&(\textsc {contains}^i_{\mathcal {S}_1, q} \wedge \textsc {contains}^i_{\mathcal {S}_2, \overline{q}}\, \wedge (\textsc {polarity}_{\mathcal {S}_1, u} \vee \textsc {polarity}_{\mathcal {S}_2, u})). \end{aligned}$$

### Lemma 5

Let $\varPhi = Q_1X_1 \dots Q_kX_k.\varphi$ be a PCNF formula and let $\mathcal {P}$ be an LDQ(D)-derivation from $\varPhi$. For each $1 \le i \le k$, each literal $\ell$, every $u\in var_\forall (\varPhi )\cap X_i$, and every truth assignment $\sigma : \bigcup _{j=1}^{i-1} X_j \rightarrow \{0, 1\}$, $\textsc {top}^i_{\mathcal {P}}$ satisfies (1), $\textsc {contains}^i_{\mathcal {P}, \ell }$ satisfies (2), and $\textsc {polarity}_{\mathcal {P}, u}$ satisfies (3).

### Proof

Let $X = X_1 \cup \dots \cup X_{i-1}$. As (1) and (2) are related, we will prove them first. We will use induction on the structure of $\mathcal {P}$, with the induction hypothesis that (1) and (2) hold. The inductive step will be carried out in two phases. In the first phase, we prove that (1) holds and in the second phase we use this additional information to prove that (2) holds as well.

1. Let $\mathcal {P}$ be an input node. By Definition 8 we have $\mathcal {P}[\sigma ] = \top$ if, and only if, $Cl(\mathcal {P})[\sigma ] = 1$. Since $\sigma$ only assigns variables in *X*, this is the case if, and only if, $\textsc {top}^i_{\mathcal {P}}[\sigma ] = 1$, so (1) holds.
2. Let $\mathcal {P}= \mathcal {P}_1 \odot _q \mathcal {P}_2$ such that (1) and (2) hold for $\mathcal {P}_1$ and $\mathcal {P}_2$. We distinguish several cases.
  - $q\notin Cl(\mathcal {P}_1[\tau ])$. Then $\mathcal {P}[\tau ]=\mathcal {P}_1[\tau ]$. Since $q\notin Cl(\mathcal {P}_1[\tau ])$, it cannot be the case that $\mathcal {P}_1[\tau ]=\top$ and so $\mathcal {P}[\tau ]\ne \top$ as well. By the induction hypothesis, we have $\textsc {contains}^i_{\mathcal {P}_1, q}[\tau ]=0$ and also $\textsc {top}^i_{\mathcal {P}_1}[\tau ]=0$ which means $\textsc {top}^i_{\mathcal {P}}[\tau ]=0$ as required.
  - $q\in Cl(\mathcal {P}_1[\tau ])$ and $\overline{q}\notin Cl(\mathcal {P}_2[\tau ])$. Then $\mathcal {P}[\tau ]=\mathcal {P}_2[\tau ]$. Since $\overline{q}\notin Cl(\mathcal {P}_2[\tau ])$, we cannot have $\mathcal {P}_2[\tau ]=\top$ and thus $\mathcal {P}[\tau ]\ne \top$ as well. By the induction hypothesis, we have $\textsc {contains}^i_{\mathcal {P}_2, \overline{q}}[\tau ]=0$ and also $\textsc {top}^i_{\mathcal {P}_2}[\tau ]=0$ which means $\textsc {top}^i_{\mathcal {P}}[\tau ]=0$ as required.
  - $q\in Cl(\mathcal {P}_1[\tau ])$ and $\overline{q}\in Cl(\mathcal {P}_2[\tau ])$ and $\mathcal {P}_1[\tau ]=\top$ or $\mathcal {P}_2[\tau ]=\top$. Then $\mathcal {P}[\tau ]=\top$ and by induction hypothesis, we have $\textsc {contains}^i_{\mathcal {P}_1, q}[\tau ] = 1$ as well as $\textsc {contains}^i_{\mathcal {P}_2, \overline{q}}[\tau ] = 1$, and $\textsc {top}^i_{\mathcal {P}_1}[\tau ]=1$ or $\textsc {top}^i_{\mathcal {P}_2}[\tau ]=1$. In any case, $\textsc {top}^i_{\mathcal {P}}[\tau ]=1$.
  - $q\in Cl(\mathcal {P}_1[\tau ])$ and $\overline{q}\in Cl(\mathcal {P}_2[\tau ])$ and $\mathcal {P}_1[\tau ]\ne \top$ and $\mathcal {P}_2[\tau ]\ne \top$. Then $\mathcal {P}[\tau ]=\mathcal {P}_1[\tau ]\odot _q\mathcal {P}_2[\tau ]\ne \top$. By induction hypothesis, we have $\textsc {top}^i_{\mathcal {P}_1}[\tau ] = 0$ and $\textsc {top}^i_{\mathcal {P}_2}[\tau ] = 0$, which ensures $\textsc {top}^i_{\mathcal {P}}[\tau ]=0$.
3. Let $\mathcal {P}= \mathcal {P}' - u$. From the definitions, we can immediately see that $\mathcal {P}'[\tau ]=\top \Longleftrightarrow \mathcal {P}[\tau ]=\top$ and $\textsc {top}^i_{\mathcal {P}'}=\textsc {top}^i_{\mathcal {P}}$ which proves (1).

We have proved that $\mathcal {P}[\tau ]=\top \Longleftrightarrow \textsc {top}^i_{\mathcal {P}}[\tau ]=1$, and it can be easily checked that, by definition, $\textsc {top}^i_{\mathcal {P}}\Rightarrow \textsc {contains}^i_{\mathcal {P}, \ell }$ for every literal $\ell$. Therefore, if $\mathcal {P}[\tau ]=\top$, (2) holds and in the following, we can restrict ourselves to the cases when $\mathcal {P}[\tau ]\ne \top$. Also, we can restrict ourselves to the cases when $\ell$ (the literal in question) actually belongs to $Cl(\mathcal {P})$, because otherwise $\textsc {contains}^i_{\mathcal {P},\ell }=\textsc {top}^i_{\mathcal {P}}$ and in that case (2) clearly holds.

1. Let $\mathcal {P}$ be an input node. We may assume $\mathcal {P}[\tau ]\ne \top$ and $\ell \in Cl(\mathcal {P})$ by the above. By definition, we can easily see that $\textsc {contains}^i_{\mathcal {P},\ell }[\tau ]=1$ if, and only if, $\ell \in Cl(\mathcal {P}[\tau ])$.
2. Let $\mathcal {P}= \mathcal {P}_1 \odot _q \mathcal {P}_2$ such that (1) and (2) hold for $\mathcal {P}_1$ and $\mathcal {P}_2$. We distinguish several cases.
  - $q\notin Cl(\mathcal {P}_1[\tau ])$. By the induction hypothesis, we have $\textsc {contains}^i_{\mathcal {P}_1,q}[\tau ]=0$. Also $\mathcal {P}[\tau ]=\mathcal {P}_1[\tau ]$ and
    $$\begin{aligned} \ell \in Cl(\mathcal {P}[\tau ])\Longleftrightarrow \ell \in Cl(\mathcal {P}_1[\tau ])\Longleftrightarrow \textsc {contains}^i_{\mathcal {P}_1,\ell }[\tau ], \end{aligned}$$
    where the second equivalence holds by induction hypothesis. Since we have $\textsc {contains}^i_{\mathcal {P}_1,q}[\tau ]=0$, we can write
    $$\begin{aligned} \textsc {contains}^i_{\mathcal {P}_1,\ell }[\tau ] \Longleftrightarrow \lnot \textsc {contains}^i_{\mathcal {P}_1,q}[\tau ] \wedge \textsc {contains}^i_{\mathcal {P}_1,\ell }[\tau ]. \end{aligned}$$
    Because $\textsc {contains}^i_{\mathcal {P}_1,q}[\tau ]=0$ and $\textsc {top}^i_{\mathcal {P}}[\tau ]=0$, the only disjunct in the definition of $\textsc {contains}^i_{\mathcal {P},\ell }[\tau ]$ that can possibly be satisfied is the second one, so that
    $$\begin{aligned} \textsc {contains}^i_{\mathcal {P},\ell }[\tau ] \Longleftrightarrow \lnot \textsc {contains}^i_{\mathcal {P}_1,q}[\tau ] \wedge \textsc {contains}^i_{\mathcal {P}_1,\ell }[\tau ], \end{aligned}$$
    which establishes (2).
  - $q\in Cl(\mathcal {P}_1[\tau ])$ and $\overline{q}\notin Cl(\mathcal {P}_2[\tau ])$. By the induction hypothesis, we have $\textsc {contains}^i_{\mathcal {P}_1,q}[\tau ]=1$ and $\textsc {contains}^i_{\mathcal {P}_2,\overline{q}}[\tau ]=0$. An argument symmetric to the one for the preceding case can be used to show (2).
  - $q\in Cl(\mathcal {P}_1[\tau ])$ and $\overline{q}\in Cl(\mathcal {P}_2[\tau ])$ and $\mathcal {P}_1[\tau ]=\top$ or $\mathcal {P}_2[\tau ]=\top$. In this case $\mathcal {P}[\tau ]=\top$ which has already been taken care of (see above).
  - $q\in Cl(\mathcal {P}_1[\tau ])$ and $\overline{q}\in Cl(\mathcal {P}_2[\tau ])$ and $\mathcal {P}_1[\tau ]\ne \top$ and $\mathcal {P}_2[\tau ]\ne \top$. Then $\mathcal {P}[\tau ]=\mathcal {P}_1[\tau ]\odot _q\mathcal {P}_2[\tau ]$ and since we have restricted ourselves to the case when $\ell \in Cl(\mathcal {P})$ (see above), we have
    $$\begin{aligned} \ell \in Cl(\mathcal {P}[\tau ])&\Longleftrightarrow \ell \in Cl(\mathcal {P}_1[\tau ])\vee \ell \in Cl(\mathcal {P}_2[\tau ])\\&\Longleftrightarrow \textsc {contains}^i_{\mathcal {P}_1,\ell }[\tau ]\vee \textsc {contains}^i_{\mathcal {P}_2,\ell }[\tau ], \end{aligned}$$
    where the final equivalence follows from the induction hypothesis. It is straightforward to verify that the last expression in turn is equivalent to the fourth disjunct in the definition of $\textsc {contains}^i_{\mathcal {P},\ell }$ being satisfied, and since this is the only disjunct that can be satisfied in this case, we conclude that (2) holds.
  By Definition 8, $\mathcal {P}[\sigma ] = \top$ if, and only if, $\mathcal {P'}[\sigma ] = \top$, and $\ell \in Cl(\mathcal {P}[\sigma ])$ if, and only if, $\ell \in Cl(\mathcal {P}'[\sigma ])$, for each literal $\ell \in Cl(\mathcal {P})$. Since (1) and (2) hold for $\mathcal {P}'$ by induction hypothesis, these properties must hold for $\mathcal {P}$ as well.

Let us now turn to the proof of (3).

1. If $\mathcal {P}$ is an input node we have
  $$\begin{aligned} u\in \mathcal {P}[\tau ]\Longleftrightarrow u\in Cl(\mathcal {P}[\tau ])\Longleftrightarrow \textsc {contains}^i_{\mathcal {P},u}[\tau ]=\textsc {polarity}_{\mathcal {P},u}[\tau ] \end{aligned}$$
  by what we proved previously and the definition of $\textsc {polarity}_{\mathcal {P},u}$ for input nodes (and the fact that a literal appears in a derivation that consists of a single input node iff it occurs in the clause of associated with that node).
2. Let $$\mathcal {P}= \mathcal {P}_1 \odot _q \mathcal {P}_2$$.
  - $q\notin Cl(\mathcal {P}_1[\tau ])$. Then $\mathcal {P}[\tau ]=\mathcal {P}_1[\tau ]$ and by the induction hypothesis, we have
    $$\begin{aligned} u\text { appears in }\mathcal {P}[\tau ]\Longleftrightarrow u\text { appears in }\mathcal {P}_1[\tau ]\Longleftrightarrow \textsc {polarity}_{\mathcal {P}_1,u}[\tau ] = 1. \end{aligned}$$
    Using (2), it is readily verified that $\textsc {polarity}_{\mathcal {P},u}[\tau ] = \textsc {polarity}_{\mathcal {P}_1,u}[\tau ]$.
  - $q\in Cl(\mathcal {P}_1[\tau ])$ and $\overline{q}\notin Cl(\mathcal {P}_2[\tau ])$. Here, (3) can be proved using an argument symmetric to one for the previous case.
  - $q\in Cl(\mathcal {P}_1[\tau ])$ and $\overline{q}\in Cl(\mathcal {P}_2[\tau ])$ and $\mathcal {P}_1[\tau ]=\top$ or $\mathcal {P}_2[\tau ]=\top$. Then $\mathcal {P}[\tau ]=\top$, so *u* appears in $\mathcal {P}[\tau ]$. Without loss of generality, let $\mathcal {P}_1[\tau ]=\top$. By the induction hypothesis we have $\textsc {polarity}_{\mathcal {P}_1,u}[\tau ]=1$, which, along with the assumptions for this case and (2), implies that $\textsc {polarity}_{\mathcal {P},u}$ is satisfied by the last disjunct.
  - $q\in Cl(\mathcal {P}_1[\tau ])$ and $\overline{q}\in Cl(\mathcal {P}_2[\tau ])$ and $\mathcal {P}_1[\tau ]\ne \top$ and $\mathcal {P}_2[\tau ]\ne \top$. In this case *u* appears in $\mathcal {P}[\tau ]$ if, and only if, it appears in $\mathcal {P}_1[\tau ]$ or in $\mathcal {P}_2[\tau ]$. Using the induction hypothesis and (2), one can verify that this is the case if, and only if, $\textsc {polarity}_{\mathcal {P}, u}[\tau ] = 1$.$\square$

These auxiliary circuits can be efficiently constructed in a top-down manner, from the input nodes to the conclusion. By a careful analysis, we obtain the following:

### Lemma 6

There is an algorithm that, given a PCNF formula $\varPhi$ and an LDQ(D)-derivation $\mathcal {P}$ from $\varPhi$, computes the circuits $\textsc {polarity}_{\mathcal {P}, u}$ for every universal variable *u* in time $O(|\mathcal {P}|\cdot n)$, where $n = | var (\varPhi )|$.

### Proof

The algorithm first sorts clauses according to a fixed order of literals. Let *k* be the number of quantifier blocks in the prefix of $\varPhi$. There is at most one circuit $\textsc {top}^i_{\mathcal {P}}$ for each node ${\mathcal {S}}$ of $\mathcal {P}$ and each $1 \le i \le k$. Similarly, there is at most one circuit $\textsc {contains}^i_{\mathcal {S}, \ell }$ for each node ${\mathcal {S}}$ of $\mathcal {P}$, each $1 \le i \le k$, and each literal $\ell \in Cl(\mathcal {S})$.

Once $\textsc {top}^i_{\mathcal {S}}$ has been computed for each $1 \le i \le k$, the circuits $\textsc {contains}^i_{\mathcal {S}, \ell }$ can easily be constructed for each $1 \le i \le k$ and every literal $\ell \in Cl(\mathcal {S})$. Overall, this can be done in time

$$\begin{aligned} O(|Cl(\mathcal {S})| \cdot k) \le O(|Cl(\mathcal {S})| \cdot n). \end{aligned}$$

Assume that the circuits $\textsc {contains}^i_{\mathcal {S}, \ell }$ are stored in lists following the order of literals in $Cl(\mathcal {S})$. Then for each node $\mathcal {S}$, the circuits $\textsc {top}^i_{\mathcal {S}}$ and $\textsc {contains}^i_{\mathcal {S}, \ell }$ associated with ${\mathcal {S}}$ can again be computed in time $O(|Cl(\mathcal {S})| \cdot n)$, so that overall, these circuits can be computed in time $O(|\mathcal {P}|\cdot n)$ for all nodes of $\mathcal {P}$. Having computed the circuits $\textsc {contains}$ and $\textsc {top}$, the circuits $\textsc {polarity}_{\mathcal {S}, u}$ can be computed for each node ${\mathcal {S}}$ and each universal variable $u \in var _{\forall }(\varPhi )$ in time $O(|\mathcal {P}| \cdot n)$. $\square$

Using Lemma 4, we can spell out the argument sketched at the beginning of this section and prove that for normal dependency schemes D, the universal player can maintain an LDQ(D)-refutation throughout the evaluation game by successively restricting an initial LDQ(D)-refutation by both players’ moves and assigning universal variables from the leftmost remaining block *X* so as to falsify the (unique) literals from *X* remaining in the refutation. Lemma 5 tells us that the $\textsc {polarity}$ circuits can be used to implement this strategy. In order to put things together, we will need the following two lemmas, which tell us that successive restriction and bulk restriction in fact yield the same result.

### Lemma 7

Let $\mathcal {P}$ be an LDQ(D)-derivation from a PCNF formula $\varPhi$, let $\tau _1$, $\tau _2$ be two assignments to disjoint sets of variables. Then $\mathcal {P}[\tau _1][\tau _2]=\mathcal {P}[\tau _1 \cup \tau _2]$.

### Proof

By induction on the structure of the derivation. If $\mathcal {P}$ is an input node, we have $Cl(\mathcal {P}[\tau ])=Cl(\mathcal {P})[\tau ]=Cl(\mathcal {P})[\tau _1][\tau _2]=Cl(\mathcal {P}[\tau _1][\tau _2])$ and since both derivations consist of a single node with the same label, they are in fact equal. For derivations created by the operations, the equality is trivially preserved. $\square$

### Lemma 8

Let D be a normal dependency scheme, let $\varPhi = Q_1 X_1 \dots Q_k X_k.\varphi$ be a PCNF formula, let $\mathcal {P}$ be an LDQ(D)-refutation of $\varPhi$. Let $X_i$ be a universal quantifier block and let $\tau : \bigcup _{j=1}^{i-1}X_j \rightarrow \{0, 1\}$ be an assignment. If $\mathcal {P}[\tau ]$ is an LDQ(D)-refutation of $\varPhi [\tau ]$, then $\mathcal {P}[\tau \cup \sigma ]$ is an LDQ(D)-refutation of $\varPhi [\tau \cup \sigma ]$, where $\sigma : X_i \rightarrow \{0, 1\}$ is the assignment such that $\sigma (u) = \lnot \textsc {polarity}_{\mathcal {P}, u}[\tau ]$ for each $u \in X_i$.

### Proof

Assume $\mathcal {P}[\tau ]$ is an LDQ(D)-refutation of $\varPhi [\tau ]$. Let $u \in X_i$. Because D is simple, variable *u* appears in $\mathcal {P}[\tau ]$ in at most one polarity. If *u* does not appear in $\mathcal {P}[\tau ]$ at all, the restriction $\mathcal {P}[\tau ][\sigma ]$ does not depend on $\sigma (u)$. Otherwise, there is a unique literal $\ell$ with $var (\ell ) = u$ that appears in $\mathcal {P}[\tau ]$. By Lemma 5, $\textsc {polarity}_{\mathcal {P}, u}[\tau ] = 1$ iff *u* appears in $\mathcal {P}[\tau ]$, so $\sigma (u) = \lnot \textsc {polarity}_{\mathcal {P}, u}[\tau ] = 0$ if $\ell = u$ and $\sigma (u) = 1$ if $\ell = \lnot u$. It is a straightforward consequence that $\mathcal {P}[\tau ][\sigma ]$ can be obtained from $\mathcal {P}[\tau ]$ by deleting every occurrence of a variable $u \in X_i$ and omitting instances of $\forall$-reduction that become redundant as a result. Because D is monotone, the restriction $\mathcal {P}[\tau ][\sigma ]$ is an LDQ(D)-refutation of $\varPhi [\tau \cup \sigma ]$, and $\mathcal {P}[\tau ][\sigma ] = \mathcal {P}[\tau \cup \sigma ]$ by Lemma 7. $\square$

With that, we are ready to prove the final statement.

### Lemma 9

Let D be a normal dependency scheme, let $\mathcal {P}$ be an LDQ(D)-refutation of a PCNF formula $\varPhi$. Then the family $\{f_u\}_{u \in var _{\forall }(\varPhi )}$ of functions $f_u = \lnot \textsc {polarity}_{\mathcal {P}, u}$ is a countermodel of $\varPhi$.

### Proof

Let $\varPhi = Q_1X_1\dots Q_kX_k.\varphi$ and let $\tau : var (\varPhi ) \rightarrow \{0, 1\}$ be a truth assignment such that $\tau (u) = f_u\left( \tau |_{L_{\varPhi }(u)}\right)$ for each universal variable *u*. Let $X_{<i} = \bigcup _{j=1}^{i-1}X_j$, and let $\tau _i = \tau |_{X_{<i}}$ for each $1 \le i \le k + 1$. We claim that $\mathcal {P}[\tau _i]$ is an LDQ(D)-refutation of $\varPhi [\tau _i]$ for $1 \le i \le k + 1$. The assignment $\tau _1$ is empty so $\mathcal {P}[\tau _1] = \mathcal {P}$ and $\varPhi [\tau _1] = \varPhi$ so the statement holds in that case. Suppose the claim holds for *i* such that $1 \le i \le k$. If $Q_{i} = \exists$, then $\mathcal {P}[\tau _{i}][\tau |_{X_i}]$ is an LDQ(D)-refutation of $\varPhi [\tau _{i+1}]$ by Lemma 4, and $\mathcal {P}[\tau _{i}][\tau |_{X_i}] = \mathcal {P}[\tau _{i+1}]$ by Lemma 7. Otherwise, $Q_i = \forall$ and $\mathcal {P}[\tau _{i+1}]$ is an LDQ(D)-refutation of $\varPhi [\tau _{i+1}]$ by Lemma 8. This completes the proof of the claim. In particular, we now have that $\mathcal {P}[\tau _{k+1}] = \mathcal {P}[\tau ]$ is an LDQ(D)-refutation of $\varPhi [\tau _{k+1}] = \varPhi [\tau ]$. Because $\varPhi [\tau ]$ does not contain any variables, the only way $\varPhi [\tau ]$ can have a refutation is that its matrix contains the empty clause, which means that $\varphi [\tau ] = \{\emptyset \}$. $\square$

Theorem 1 follows immediately from Lemmas 6 and  9. In fact, we obtain the following result.

### Theorem 3

Let *D* be a normal dependency scheme. There is an algorithm that computes a countermodel of a PCNF formula $\varPhi$ with *n* variables from an LDQ(D)-refutation $\mathcal {P}$ of $\varPhi$ in time $O(|\mathcal {P}|\cdot n)$.

## Footnotes

1 We consider QBFs in prenex normal form.

2 The original definition of dependency schemes [39] is more restrictive than the one given here, but the additional requirements are irrelevant for the purposes of this paper.

3 Strictly speaking, it uses a refined version of the standard dependency scheme [31, p. 49].

4 The resolution rule as defined here is more general than the one considered in an earlier version of this paper [36], in that we admit complementary universal literals to be “merged” as long as the pivot is independent according to D (rather than $\text {D}^{\text { trv}}$). This definition—which we is required to capture proofs generated by DepQBF (see Example 1 in Sect. 4)—was proposed in (independent) work by Beyersdorff and Blinkhorn [6].

5 The presentation is adapted from a recent paper on QCDCL with *dependency learning* [37].

6 For simplicity, we do not consider the pure literal rule as part of QBCP.

7 http://lonsing.github.io/depqbf/.

8 As a sanity check, we verified that all configurations that were able to solve a particular instance returned the same result.

9 http://fmv.jku.at/bloqqer/.
